# On asymptotic joint distributions of cherries and pitchforks for random phylogenetic trees

**DOI:** 10.1007/s00285-021-01667-2

**Published:** 2021-09-23

**Authors:** Kwok Pui Choi, Gursharn Kaur, Taoyang Wu

**Affiliations:** 1 Department of Statistics and Data Science, and the Department of Mathematics, National University of Singapore, Singapore, 117546 Republic of Singapore; 2 School of Computing Sciences, University of East Anglia, Norwich, NR4 7TJ UK

**Keywords:** Tree shape, Joint subtree distributions, Pólya urn model, Limit distributions, Yule-Harding-Kingman model, PDA model, 92B10, 60F05, 92D99

## Abstract

Tree shape statistics provide valuable quantitative insights into evolutionary mechanisms underpinning phylogenetic trees, a commonly used graph representation of evolutionary relationships among taxonomic units ranging from viruses to species. We study two subtree counting statistics, the number of cherries and the number of pitchforks, for random phylogenetic trees generated by two widely used null tree models: the proportional to distinguishable arrangements (PDA) and the Yule-Harding-Kingman (YHK) models. By developing limit theorems for a version of extended Pólya urn models in which negative entries are permitted for their replacement matrices, we deduce the strong laws of large numbers and the central limit theorems for the joint distributions of these two counting statistics for the PDA and the YHK models. Our results indicate that the limiting behaviour of these two statistics, when appropriately scaled using the number of leaves in the underlying trees, is independent of the initial tree used in the tree generating process.

## Introduction

As a common mathematical representation of evolutionary relationships among biological systems ranging from viruses to species, phylogenetic trees retain important signatures of the underlying evolutionary events and mechanisms which are often not directly observable, such as rates of speciation and expansion (Mooers et al. 2007; Heath et al. 2008). To utilise these signatures, one popular approach is to compare empirical shape indices computed from trees inferred from real datasets with those predicted by neutral models specifying a tree generating process  (see, e.g. Blum and François 2006; Hagen et al. 2015). Moreover, topological tree shapes are also informative for understanding several fundamental statistics in population genetics (Ferretti et al. 2017; Arbisser et al. 2018) and important parameters in the dynamics of virus evolution and propagation (Colijn and Gardy 2014).

This paper focuses on two subtree counting statistics: the number of cherries (i.e., nodes that have precisely two descendent leaves) and that of pitchforks (i.e., nodes that have precisely three descendent leaves) in a tree. These statistics are related to monophylogenetic structures in phylogenetic trees (Rosenberg 2003) and have been utilised recently to study evolutionary dynamics of pathogens (Colijn and Gardy 2014). For example, the asymptotic frequency of cherries in pathogen trees generated by some models can be used to estimate the basic reproduction number (Plazzotta and Colijn 2016) and to study the impact of the underlying contact network over which a pathogen spreads (Metzig et al. 2019). Various properties concerning these statistics have been established in the past decades on the following two fundamental random phylogenetic tree models: the Yule-Harding-Kingman (YHK) (Rosenberg 2006; Disanto and Wiehe 2013; Holmgren and Janson 2015) and the proportional to distinguishable arrangements (PDA) models (McKenzie and Steel 2000; Chang and Fuchs 2010; Wu and Choi 2016; Choi et al. 2020).

In this paper we are interested in the limiting behaviour of the joint cherry and pitchfork distributions for the YHK and the PDA models. In a seminal paper, McKenzie and Steel (2000) showed that cherry distributions converge to a normal distribution, which was later extended to pitchforks and other subtrees by Chang and Fuchs (2010). More recently, Holmgren and Janson (2015) studied subtree counts in the random binary search tree model, and their results imply that the cherry and pitchfork distributions converge jointly to a bivariate normal distribution under the YHK model. This is further investigated by Wu and Choi (2016) and Choi et al. (2020), where numerical results indicate that convergence to bivariate normal distributions holds under both the YHK model and the PDA model. Our main results, Theorems 1 and 2, provide a unifying approach to establishing the convergence of the joint distributions to bivariate normal distributions for both models, as well as a strong law stating that the joint counting statistics converge almost surely (a.s.) to a constant vector. Moreover, our results indicate that the limiting behaviour of these two statistics, when appropriately scaled, is independent of the initial tree used in the tree generating process.

Our approach is based on a general model in probability theory known as the Pólya urn scheme, which has been developed during the past few decades including applications in studying various growth phenomena with an underlying random tree structure (see, e.g. Mahmoud (2009) and the references therein). For instance, the results by McKenzie and Steel (2000) are based on a version of the urn model in which the off-diagonal elements in the replacement matrix are all positive. However, such technical constraints pose a central challenge for studying pitchfork distributions as negative entries in the resulting replacement matrix are not confined only to the diagonal (see Sects. 4 and 5). To overcome this limitation, we study a family of extended Pólya urn models under certain technical assumptions in which negative entries are allowed for their replacement matrices (see Sect. 3). Inspired by the martingale approach used by Bai and Hu (2005), we present a self-contained proof for the limit theorems for this extended urn model, with the dual aims of completeness and accessibility. Our approach is different from a popular framework in which discrete urn models are embedded into a continuous Markov chain known as the branching processes (see, e.g. Janson (2004) and the references therein).

We summarize the contents of the rest of the paper. In the next section, we collect some definitions concerning phylogenetic trees and the two tree-based Markov processes. In Sect. 3, we introduce the urn model and a version of the Strong Law of Large Numbers and the Central Limit Theorem that are applicable to our study. We apply these two theorems to the YHK process in Sect. 4, and the PDA process in Sect. 5. These results are then extended to unrooted trees in Sect. 6. The proofs of the main results for the urn model are presented in Sect. 7, with a technical lemma included in the appendix. We conclude this paper in the last section with a discussion of our results and some open problems.

## Preliminaries

In this section, we present some basic notation and background concerning phylogenetic trees, random tree models, and urn models. Throughout this paper, *n* is a positive integer greater than two unless stated otherwise.

### Phylogenetic trees

A *tree* $T=\left( V(T),E(T) \right)$ is a connected acyclic graph with vertex set *V*(*T*) and edge set *E*(*T*). A vertex is referred to as a *leaf* if it has degree one, and an *interior vertex* otherwise. An edge incident to a leaf is called a *pendant edge*, and let $E^{\circ }(T)$ be the set of pendant edges in *T*. A tree is *rooted* if it contains exactly one distinguished degree one node designated as the *root*, which is not regarded as a leaf and is usually denoted by $\rho$, and *unrooted* otherwise. Moreover, the orientation of a rooted tree is from its root to its leaves. Other than those in Sect. 6, all trees considered in this paper are rooted and *binary*, that is, each interior vertex has precisely two children.

**Fig. 1:**
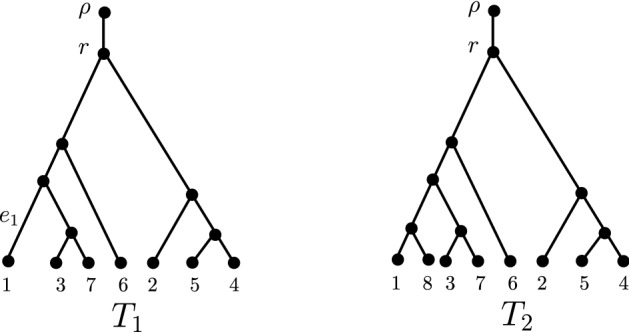
Examples of phylogenetic trees. $T_1$ is a rooted phylogenetic tree on $\{1,\dots ,7\}$; $T_2=T_1[e_{1}]$ is a phylogenetic tree on $X=\{1,\dots ,8\}$ obtained from $T_1$ by attaching a new leaf labelled 8 to the edge $e_{1}$ which is incident with taxon 1 in $T_1$

A *phylogenetic tree* on a finite set *X* is a rooted binary tree with leaves bijectively labelled by the elements of *X*. The set of binary rooted phylogenetic trees on $\{1,2,\dots ,n\}$ is denoted by ${\mathcal {T}}_n$. See Fig. 1 for examples of trees in ${\mathcal {T}}_7$ and ${\mathcal {T}}_8$. Given an edge *e* in a phylogenetic tree *T* on *X* and a taxon $x' \not \in X$, let $T[e;x']$ be the phylogenetic tree on $X\cup \{x'\}$ obtained by attaching a new leaf with label $x'$ to the edge *e*. Formally, let $e=(u,v)$ and let *w* be a vertex not contained in *V*(*T*). Then $T[e;x']$ has vertex set $V(T) \cup \{x',w\}$ and edge set $\big (E(T) \setminus \{e\} \big ) \cup \{(u,w), (w,v), (w,x')\}$. See Fig. 1 for an illustration of this construction, where tree $T_2=T_1[e_{1};8]$ is obtained from $T_1$ by attaching leaf 8 to the edge $e_{1}$. We simply use *T*[*e*] instead of $T[e;x']$ when the taxon name $x'$ is not essential.

Removing an edge in a phylogenetic tree *T* results in two connected components; the connected component that does not contain the root of *T* is referred to as a subtree of *T*, also commonly known as a fringe subtree. A subtree is called a *cherry* if it has two leaves, and a *pitchfork* if it has three leaves. Following the notation by Choi et al. (2020), let $A(T)$ and $B(T)$ be the number of pitchforks and cherries contained in *T*. For example, in Fig. 1 we have $A(T_2)=1$ and $B(T_2)=3$.

### The YHK and the PDA processes

Let ${\mathcal {T}}_n$ be the set of phylogenetic trees with *n* leaves. In this subsection, we introduce the two tree-based Markov processes investigated in this paper: the proportional to distinguishable arrangements (PDA) process and the Yule-Harding-Kingman (YHK) process. Our description of these two processes is largely based on that in Choi et al. (2020), which is adapted from the Markov processes as described by Steel (2016, Section 3.3.3).

Under the YHK process (Yule 1925; Harding 1971), starting with a given tree $$T_m$$ in $${\mathcal {T}}_m$$ with $$m\ge 2$$, a random phylogenetic tree $$T_n $$ in $${\mathcal {T}}_n$$ is generated as follows.

(i) Select a uniform random permutation $(x_1,\dots ,x_n)$ of $\{1,2,\dots ,n\}$;
(ii) label the leaves of the rooted phylogenetic tree $T_m$ randomly using the taxon set $\{x_1,x_2,\ldots ,x_m\}$;
(iii) for $m\le k <n$, uniformly choose a random pendant edge *e* in $T_k$ and let $T_{k+1}=T_k[e;x_{k+1}]$.

The PDA process can be described using a similar scheme; the only difference is that in Step (iii) the edge *e* is uniformly sampled from the edge set of $T_k$, instead of the pendant edge set. Furthermore, under the PDA process, Step (i) can also be simplified by using a fixed permutation, say $(1,2,\ldots ,n)$. In the literature, the special case $m=2$, for which $T_2$ is the unique tree with two leaves, is also referred to as the YHK model and the PDA model, respectively.

For $n\ge 4$, let $A_n$ and $B_n$ be the random variables $A(T)$ and $B(T)$, respectively, for a random tree *T* in ${\mathcal {T}}_n$. The probability distributions of $A_n$ (resp. $B_n$) are referred to as pitchfork distributions (resp. cherry distributions). In this paper, we are mainly interested in the limiting distributional properties of $(A_n, B_n)$.

### Modes of convergence

Let $X, X_1,X_2,\dots$ be random variables on some probability space $(\varOmega ,{{{\mathcal {F}}}},{\mathbb {P}})$. To study the urn model we will use the following four modes of convergence (see, e.g. Grimmett and Stirzaker (2001, Section 7.2) for more details). First, $X_n$ is said to converge to *X* *almost surely*, denoted as $X_n \xrightarrow {~a.s.~}X$, if $\{\omega \in \varOmega \,:\, X_n(\omega )\rightarrow X(\omega )~\text{ as }~n \rightarrow \infty \}$ is an event with probability 1. Next, $X_n$ is said to converge to *Xin r-th norm*, where $r>0$, written $X_n \xrightarrow {~r~}X$, if ${\mathbb {E}}(|X_n^r|)<\infty$ for all *n* and ${\mathbb {E}}(|X_n-X|^r) \rightarrow 0$ as $n\rightarrow \infty$. Furthermore, $X_n$ is said to converge to *X* *in probability*, written $X_n \xrightarrow {~p~}X$, if ${\mathbb {P}}(|X_n-X| > \epsilon ) \rightarrow 0$ as $n\rightarrow \infty$ for all $\epsilon >0$. Finally, $X_n$ converges to a random variable *Y* *in distribution*, also termed *weak convergence* or *convergence in law* and written $X_n \xrightarrow {~d~} Y$, if ${\mathbb {P}}(X_n \le x) \rightarrow {\mathbb {P}}(Y \le x)$ as $n\rightarrow \infty$ for all points *x* at which the distribution function ${\mathbb {P}}(Y\le x)$ is continuous. Note that $X_n \xrightarrow {~p~}X$ implies $X_n \xrightarrow {~d~} X$, and $X_n \xrightarrow {~p~}X$ holds if either $X_n \xrightarrow {~a.s.~}X$ holds or $X_n\xrightarrow {~r~}X$ holds for some $r >0$.

### Miscellaneous

Let ${\mathbf {0}}=(0,\dots ,0)$ be the $d$-dimensional zero row vector. Let ${\mathbf {e}}=(1,\dots ,1)$ be the $d$-dimensional row vector whose entries are all one, and for $1 \le j \le d$, let ${\mathbf{e}}_j$ denote the *j*-th canonical row vector whose *j*-th entry is 1 while the other entries are all zero.

Let $\text {diag}(a_1,\dots ,a_d)$ denote a diagonal matrix whose diagonal elements are $a_1, \ldots , a_d$. Furthermore, ${\mathbf {0}}^\top {\mathbf {0}}$ is the $d\times d$ matrix whose entries are all zero. Here $Z^\top$ denotes the transpose of *Z*, where *Z* can be either a vector or a matrix.

## Urn models

In this section, we briefly recall the classical Pólya urn model and some of its generalisations. Pólya urn model was studied by Pólya (1930) and can be traced back to Markov (see, e.g. Johnson and Kotz (1977, Section 1.2)). It has been applied to describe evolutionary processes in biology and computer science. Several such applications in genetics are discussed by Johnson and Kotz (1977, Chapter 5) and by Mahmoud (2009, Chapters 8 and 9). In a general setup, consider an urn with balls of $$d$$ different colours containing $$C_{0,i}$$ many balls of colour $$i \in \{1,2,\dots , d\}$$ at time 0. At each time step, a ball is drawn uniformly at random and returned with some extra balls, depending on the colour selected. The reinforcement scheme is often described by a $$d\times d$$ matrix *R*: if the colour of the ball drawn is *i*, then we return the selected ball along with $$R_{ij} $$ many balls of colour *j*, for every $$j \in \{ 1,2,\dots , d\}$$, where a positive value of $$R_{ij}$$ means adding $$R_{ij}$$ balls and a negative value of $$R_{ij}$$ means removing $$|R_{ij}|$$ many balls from the urn. Such a matrix is termed as *replacement matrix* in the literature. For instance, the replacement matrix *R* is the identity matrix for the original Pólya urn model with *d* colours: at each time point, the selected ball is returned with one additional ball of the same colour. We restrict our attention to tenable urn processes, that is, at each step it is always possible to add or remove balls according to the matrix *R*.

Let $C_n = (C_{n,1}, \dots , C_{n,d})$ be the row vector of dimension $d$ that represents the ball configuration at time *n* for an urn model with $d$ colours, in which each entry is necessarily non-negative and at least one of these entries is greater than 0. Then the sum of $C_{n,i}$, denoted by $t_n$, is the number of balls in the urn at time *n*. Note that throughout this paper, $t_n$ is always a number greater than 0. Recall that a vector is referred to as a *stochastic vector* if each entry in the vector is a non-negative real number and the sum of its entries is one. Denote the stochastic vector associated with $C_n$ by ${\widetilde{C}}_n$, that is, we have ${\widetilde{C}}_{n,i}=C_{n,i}/t_n$ for $1\le i \le d$.

Let ${{{\mathcal {F}}}}_{n}$ be the information of the urn’s configuration from time 0 up to *n*, that is, the $\sigma$-algebra generated by $C_0,C_1,\dots ,C_n$. Let *R* denote the replacement matrix. Then, for every $n\ge 1$,

1 $$\begin{aligned} C_{n} = C_{n-1} +\chi _{n} R, \end{aligned}$$

where $\chi _{n}$ is a random row vector of length *d* such that for $i=1, \ldots , d$,

$$\begin{aligned} {\mathbb {P}}(\chi _{n} = {{\mathbf {e}}}_i \vert {{{\mathcal {F}}}}_{n-1}) = {\widetilde{C}}_{n-1,i}. \end{aligned}$$

Since precisely one entry in $\chi _{n}$ is 1 and all others are 0, it follows that

2 $$\begin{aligned} {\mathbb {E}}[\chi _n\vert {{{\mathcal {F}}}}_{n-1}]={\widetilde{C}}_{n-1} ~\quad ~\text{ and }~\quad ~ {\mathbb {E}}[\chi _n^\top \chi _n\vert {{{\mathcal {F}}}}_{n-1}]=\text {diag}({\widetilde{C}}_{n-1}). \end{aligned}$$

We state the following assumptions about the replacement matrix *R*:

1. *Tenable:* It is always possible to draw balls and follow the replacement rule, that is, we never get stuck in following the rules (see, e.g. Mahmoud (2009, p.46)).
2. *Small:* All eigenvalues of *R* are real; the maximal eigenvalue $\lambda _1=s$ is positive with $\lambda _1>2\lambda$ holds for all other eigenvalues $\lambda$ of *R*.
3. *Strictly Balanced:* The column vector ${\mathbf {e}}^\top$ is a right eigenvector of *R* corresponding to $\lambda _1$ and one of the left eigenvectors corresponding to $\lambda _1$ is a stochastic vector. Note that ${\mathbf {e}}^\top$ being a right eigenvector implies $t_n = t_0+ns$, and hence the urn models discussed here are *balanced*, as commonly known in the literature.
4. *Diagonalisable:* *R* is diagonisable over real numbers. That is, there exists an invertible matrix *U* with real entries such that
  3 $$\begin{aligned} U^{-1}RU = \text {diag}(\lambda _1, \lambda _2,\dots , \lambda _d) =: \varLambda , \end{aligned}$$
  where $\lambda _1\ge \lambda _2 \ge \dots \ge \lambda _d$ are all eigenvalues of *R*.

For the matrix *U* in (A4) and $1\le j \le d$, let ${\mathbf {u}}_j=U{{\mathbf {e}}}^\top _j$ denote the *j*-th column of *U*, and ${\mathbf {v}}_j={{\mathbf {e}}}_jU^{-1}$ the *j*-th row of $U^{-1}$. Then ${\mathbf {u}}_j$ and ${\mathbf {v}}_j$ are, respectively, *right and left eigenvectors* corresponding to $\lambda _j$. Furthermore, since ${\mathbf {v}}_i {\mathbf {u}}_j={{\mathbf {e}}}_iU^{-1} U{\mathbf{e}}^\top _j ={{\mathbf {e}}}_i {\mathbf {I}}\, {{\mathbf {e}}}^\top _j$, where ${\mathbf {I}}$ is the identity matrix, we have

4 $$\begin{aligned} {\mathbf {v}}_i {\mathbf {u}}_j=1~~\text{ if } i=j, \text{ and } {\mathbf {v}}_i {\mathbf {u}}_j=0~~\text{ if } i\not =j. \end{aligned}$$

In view of (A3), (A4) and (4), for simplicity the following convention is used throughout this paper:

5 $$\begin{aligned} {\mathbf {u}}_1={\mathbf {e}}^\top ~\quad \text{ and }~~\quad {\mathbf {v}}_1~\text{ is } \text{ a } \text{ stochastic } \text{ vector }. \end{aligned}$$

Furthermore, the eigenvalue $\lambda _1$ is referred to as the *principal eigenvalue*; ${\mathbf {u}}_1$ and ${\mathbf {v}}_1$ specified in  (5) as the *principal right and principal left eigenvector*, respectively.

Motivated by adaptive clinical trial problems, Bai and Hu (2005) derived limit results in an urn model by martingale techniques. Moreover, they considered random replacement matrices but required the replacement matrix has non-negative elements. On the other hand, the limit results derived in Janson (2004) are based on an embedding of the urn model into a continuous time branching process under certain non-trivial technical assumptions of the associated continuous time branching process. In this paper, we prove first and second order limit results for an urn model with a replacement matrix that may contain non-negative elements. As mentioned earlier, our proofs are based on the martingale approach for the urn models used by Bai and Hu (2005). Under assumptions (A1)-(A4), the exact expression for the limiting variance matrix agrees with the one obtained by Bai and Hu (2005) and by Janson (2004). Notice that the assumption of real eigenvalues in (A2) and real eigenvectors in (A4) is chosen to make our proof more accessible to a wider audience by simplifying expressions and the proofs. Indeed, our proof can be extended to the case where the dominating eigenvalue $\lambda _1$ is real while the eigenvalues $\lambda _2, \dots , \lambda _d$ are complex-valued whose real parts are less than $\lambda _1/2$, as one of the cases studied in Janson (2004).

The limit of the urn process and the rate of convergence to the limiting vector depends on certain spectral properties of matrix *R* (see, e.g. Janson (2004) or Bai and Hu (2005)). In our context, it suffices to consider the extended Pólya urn model under the aforementioned assumptions (A1)–(A4), for which Theorems 1 and 2 below give the Strong Law of Large Numbers and the Central Limit Theorem. Our proofs, which are adapted from that of Bai and Hu (2005), are presented in Sect. 7 .

### Theorem 1

Under assumptions *(A1)–(A4)*, we have

6 $$\begin{aligned} (ns)^{-1} C_n \xrightarrow {~a.s.~}{\mathbf {v}}_1 ~\quad ~\text{ and } ~\quad ~ (ns)^{-1} C_n \xrightarrow {~r~}{\mathbf {v}}_1 ~\quad ~\text{ for } r >0, \end{aligned}$$

where $s$ is the principal eigenvalue and ${\mathbf {v}}_1$ is the principal left eigenvector.

Let ${\mathcal {N}}({\mathbf {0}}, \varSigma )$ be the multivariate normal distribution with mean vector ${\mathbf {0}}$ and covariance matrix $\varSigma$.

### Theorem 2

Under assumptions *(A1)–(A4)*, we have

$$\begin{aligned} n^{-1/2} (C_n - ns{\mathbf {v}}_1) \xrightarrow {~d~} {{{\mathcal {N}}}}({\mathbf {0}}, \varSigma ), \end{aligned}$$

where $s$ is the principal eigenvalue, ${\mathbf {v}}_1$ is the principal left eigenvector, and

7 $$\begin{aligned} \varSigma = \sum _{i,j=2}^d \frac{s\lambda _i \lambda _j {{\mathbf {u}}}_i^\top \text{ diag }({\mathbf {v}}_1) {{\mathbf {u}}}_j }{s-\lambda _i -\lambda _j} {\mathbf {v}}_i^\top {\mathbf {v}}_j. \end{aligned}$$

### Remark 1

During the reviewing process of this paper, a reviewer suggested that an alternative approach to establishing Theorems 1 and 2 might be based on Janson (2004, Theorems 3.21 & 3.22 and Remark 4.2) and a result on the super-critical Galton-Watson process (see, e.g. Athreya and Ney (1972, Theorem 2(i) in Section III.7)), which could potentially lead to a stronger version of the results presented here.

## Limiting distributions under the YHK model

A cherry is said to be *independent* if it is not contained in any pitchfork, and *dependent* otherwise. Similarly, a pendant edge is *independent* if it is contained in neither a pitchfork nor a cherry. In this section, we study the limiting joint distribution of the random variables $A_n$ (i.e., the number of pitchforks) and $B_n$ (i.e., the number of cherries) under the YHK model.

To study the joint distribution of cherries and pitchforks, we extend the urn models used in McKenzie and Steel (2000) (see also Steel (2016, Section 3.4)) as follows. Each pendant edge in a phylogenetic tree is designated as one of the following four types:

1. a type 1 edge is a pendant edge in a dependent cherry (i.e., contained in both a cherry and a pitchfork);
2. a type 2 edge is a pendant edge in an independent cherry (i.e., contained in a cherry but not a pitchfork);
3. a type 3 edge is a pendant edge contained in a pitchfork but not a cherry;
4. a type 4 edge is an independent pendant edge (i.e., contained in neither a pitchfork nor a cherry).

**Fig. 2:**
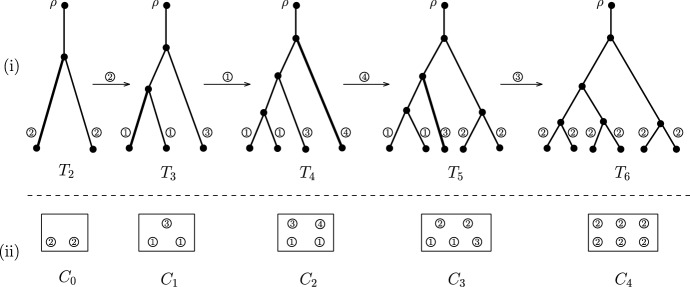
A sample path of the YHK model and the associated urn model. (i): A sample path of the YHK model evolving from $T_2$ with two leaves to $T_6$ with six leaves. The labels of the leaves are omitted for simplicity. The type of pendant edges is indicated by the circled numbers next to them. For $2\le i \le 5$, the edge selected in $T_i$ to generate $T_{i+1}$ is highlighted in bold and the associated edge type is indicated in the circled number above the arrows. (ii) The associated urn model with four colours, derived from the types of pendants edges in the trees. Note that in the vector form we have $C_0=(0,2,0,0), C_1=(2,0,1,0), C_2=(2,0,1,1), C_3=(2,2,1,0)$, and $C_4=(0,6,0,0)$

It is straightforward to see that any pendant edge in a phylogenetic tree with at least two leaves belongs to one and only one of the above four types. Furthermore, the numbers of pitchforks and independent cherries in a tree are precisely half of the numbers of type 1 and type 2 edges, respectively.

As illustrated in Fig. 2, the composition of the types of the pendant edges in *T*[*e*], the tree obtained from *T* by attaching an extra leaf to a pendant edge *e*, is determined by the composition of pendant edge types in *T* and the type of *e* as follows. When *e* is type 1, then the number of type 4 edges in *T*[*e*] increases by one compared with that in *T* while the number of edges of each of the other three types is the same. This holds because both *T*[*e*] and *T* have the same number of cherries and that of pitchforks (see $T_3$ and $T_4$ in Fig. 2). When *e* is of type 2, then the number of type 2 edges decreases by two while the numbers of type 1 and of type 3 increase by two and one, respectively. This is because in this case one independent cherry is replaced by one pitchfork (see $T_2$ and $T_3$ in Fig. 2). When *e* is type 3, one pitchfork is replaced by two independent cherries, hence the number of type 2 edges increases by four while the numbers of edges of type 1 and of type 3 decrease by two and one, respectively (see $T_5$ and $T_6$ in Fig. 2). Finally, when *e* is type 4, one independent pendant edge is replaced by one independent cherry, and hence the number of type 2 edges increases by two and that of type 4 edges decreases by one (see $T_4$ and $T_5$ in Fig. 2).

Using the dynamics described in the last paragraph, we can associate a YHK process starting with a tree $T_m$ with a corresponding urn process $(C_0,R)$ as follows. The urn model contains four colours in which colour *i* ($1\le i \le 4$) is designated for type *i* edges. In the initial urn $C_0=(C_{0,1},\dots ,C_{0,4})$, the number $C_{0,i}$ is precisely the number of type *i* edges in $T_m$. Furthermore, the replacement matrix *R* is the following $4 \times 4$ matrix:

8 $$\begin{aligned} R = \left[ \begin{array}{rrrr} 0 ~&{} 0 ~&{}0~&{}1\\ 2~&{}-2~&{}1~&{}0\\ -2~&{}4~&{}-1~&{}0\\ 0~&{}2~&{}0~&{}-1 \end{array}\right] . \end{aligned}$$

Given an arbitrary tree *T*, let $\alpha (T)=\big (|E_1(T)|, |E_2(T)|,|E_3(T)|,|E_4(T)|\big )$ be the pendant type vector associated with *T* where $|E_i(T)|$ counts the number of type *i* edges in *T* for $1\le i \le 4$.

The following result will enable us to obtain the joint distribution on pitchforks and cherries for the YHK model. Moreover, it also implies that the asymptotic behaviour of these two statistics, when appropriately scaled, is independent of the initial tree used in the YHK process.

### Theorem 3

Suppose that $T_m$ is an arbitrary phylogenetic tree with *m* leaves with $m\ge 2$, and that $T_n$ is a tree with *n* leaves generated by the YHK process starting with $T_m$. Then we have

9 $$\begin{aligned} \frac{\alpha (T_n)}{n} \xrightarrow {~a.s.~}{\mathbf {v}}_1 ~\quad ~\text{ and }~\quad ~ \frac{ \alpha (T_n) - n{\mathbf {v}}_1 }{\sqrt{n}} \xrightarrow {~d~} {\mathcal {N}}\left( {\mathbf {0}},\varSigma \right) , \end{aligned}$$

where $${\mathbf {v}}_1=\big (\frac{1}{3},\frac{1}{3},\frac{1}{6}, \frac{1}{6} \big )$$ and

10 $$\begin{aligned} \varSigma = \frac{1}{1260} \left[ \begin{array}{rrrr} \,276 ~&{}~ -388 ~&{}~ 138 ~&{}~ -26\,\\ \,-388 ~ &{}~ 724 ~&{}~ -194 ~&{}~ -142\, \\ \,138 ~&{}~ -194 ~&{}~ 69 ~&{}~ -13 \,\\ \, -26 ~&{}~ -142 ~&{}~ -13 ~&{}~ 181 \,\end{array}\right] . \end{aligned}$$

### Proof

Consider the YHK process $\{T_{n}\}_{n\ge m}$ starting with $T_m$. Let $C_{k}=\alpha (T_{k+m})$ for $k\ge 0$. Then $C_k = (C_{k,1}, \dots , C_{k,4})$, where $C_{k,i}=|E_i(T_{m+k})|$ for $1\le i \le 4$, is the urn model of 4 colours derived from the pendant edge decomposition of the YHK process. Therefore, it is a tenable model with $C_0=\alpha (T_m)$ and replacement matrix *R* as given in (8).

Note that *R* is diagonalisable as

$$\begin{aligned}U^{-1}R U=\varLambda \end{aligned}$$

holds with

11 $$\begin{aligned}&U = \left[ \begin{array}{rrrr} 1 &{} 1&{}-1&{}-1\\ 1&{}0&{}-1&{}-3\\ 1&{}-2&{}2&{}5\\ 1&{}0&{}2&{}3 \end{array}\right] , \qquad \varLambda = \left[ \begin{array}{rrrrr} 1&{}0&{}0 &{} 0 \\ 0&{}0&{}0&{}0&{} \\ 0&{}0 &{}-2&{}0 \\ 0&{}0&{}0&{}-3 \end{array}\right] \nonumber \\&\qquad \text {and } \qquad U^{-1}= \frac{1}{6} \left[ \begin{array}{rrrr} 2&{}2&{} 1&{}1\\ 2&{}-2&{}-2&{}2\\ -4&{}2&{}-2&{}4\\ 2&{}-2&{}1&{}-1 \end{array}\right] . \end{aligned}$$

Therefore, *R* satisfies condition (A4). Next, (A2) holds because *R* has eigenvalues

$$\begin{aligned} s=\lambda _1=1,~\quad ~ \lambda _2=0, ~\quad ~\lambda _3=-2, ~\quad ~\lambda _4=-3, \end{aligned}$$

where $s=\lambda _1=1$ is the principal eigenvalue. Furthermore, put ${\mathbf {u}}_i=U{\mathbf {e}}^\top _i$ and ${\mathbf {v}}_i={\mathbf {e}}_iU^{-1}$ for $1\le i \le 4$. Then (A3) follows by noting that ${\mathbf {u}}_1=(1,1,1,1)^\top$ is the principal right eigenvector, and ${\mathbf {v}}_1=\frac{1}{6}\big (2,2,1,1\big )$ is the principal left eigenvector.

Since (A1)–(A4) are satisfied by the replacement matrix *R*, by Theorem 1 it follows that

$$\begin{aligned} \frac{C_k}{k} \xrightarrow {~a.s.~}{\mathbf {v}}_1 ~~\text{ with }~~ {k\rightarrow \infty } \end{aligned}$$

and hence

$$\begin{aligned} \frac{\alpha (T_n)}{n}=\frac{n-m}{n} \frac{C_{n-m}}{n-m} \xrightarrow {~a.s.~}{\mathbf {v}}_1 ~~\text{ with }~~ {n\rightarrow \infty }.\end{aligned}$$

By Theorem 2 we have

12 $$\begin{aligned} \dfrac{ C_k - k v_1}{\sqrt{k}} \xrightarrow {~d~} {\mathcal {N}}({\mathbf {0}}, \varSigma ) ~~\text{ with }~~ {k\rightarrow \infty }, \end{aligned}$$

where

13 $$\begin{aligned} \varSigma = \sum _{i,j=2}^4 \frac{ \lambda _i \lambda _j {{\mathbf {u}}}_i^\top \text{ diag }({\mathbf {v}}_1) {{\mathbf {u}}}_j }{1-\lambda _i -\lambda _j} {\mathbf {v}}_i^\top {\mathbf {v}}_j. \end{aligned}$$

Therefore, we have

$$\begin{aligned} \frac{ \alpha (T_n) - n{\mathbf {v}}_1 }{\sqrt{n}}&=\frac{ C_{n-m} - (n-m){\mathbf {v}}_1 }{\sqrt{n}}+\frac{m{\mathbf {v}}_1}{\sqrt{n}} \\&=\frac{\sqrt{n-m}}{\sqrt{n}}\frac{ C_{n-m} - (n-m){\mathbf {v}}_1 }{\sqrt{n-m}} +\frac{m{\mathbf {v}}_1}{\sqrt{n}} \\&\xrightarrow {~d~} {\mathcal {N}}\left( {\mathbf {0}},\varSigma \right) . \end{aligned}$$

Here the convergence follows from (12) and the fact that $\frac{\sqrt{n-m}}{\sqrt{n}}$ converges to 1 and $\frac{m{\mathbf {v}}_1}{\sqrt{n}}$ converges to 0 when *n* approaches infinity. $\square$

By Theorem 3, it is straightforward to obtain the following result on the joint distribution of cherries and pitchforks, which also follows from a general result by Holmgren and Janson (2015, Theorem 1.22).

### Corollary 1

Under the YHK model, for the joint distribution $(A_n,B_n)$ of pitchforks and cherries we have

14 $$\begin{aligned} \frac{1}{n}(A_n, B_n) \xrightarrow {~a.s.~}\Big (\frac{1}{6}, \frac{1}{3} \Big ) \end{aligned}$$

and

15 $$\begin{aligned} \frac{ (A_n, B_n) - n (1/6, 1/3)}{\sqrt{n}} \xrightarrow {~d~} {\mathcal {N}}\left( {\mathbf {0}},\frac{1}{1260 } \left[ \begin{array}{rr} 69 ~&{} -28\\ -28 ~&{} 56 \end{array}\right] \right) . \end{aligned}$$

### Proof

Consider the YHK process $\{T_{n}\}_{n\ge 2}$ starting with a tree $T_2$ with two leaves. Denote the *i*-th entry in $\alpha (T_n)$ by $\alpha _{n,i}$ for $1\le i \le 4$. Then the corollary follows from Theorem 3 by noting that we have $A_n=\frac{\alpha _{n,1}}{2}$ and $B_n=\frac{\alpha _{n,1}+\alpha _{n,2}}{2}$. $\square$

The above result is consistent with the previously known results on the mean and (co-)variance of the joint distribution of cherries and pitchforks (see, e.g., Wu and Choi (2016); Choi et al. (2020)), namely, under the YHK model and for $n\ge 7$ we have

$$\begin{aligned} {\mathbb {E}}(A_n)=\frac{n}{6}, \quad {\mathbb {E}}(B_n)=\frac{n}{3}, \quad {\mathbb {V}}(A_n)=\frac{23n}{420}, \quad {\mathbb {V}}(B_n)=\frac{2n}{45},~~\text{ and }~~ Cov(A_n,B_n)=-\frac{n}{45}. \end{aligned}$$

## Limiting distributions under the PDA model

In this section, we study the limiting joint distribution of the random variables $A_n$ (i.e., the number of pitchforks) and $B_n$ (i.e., the number of essential cherries) under the PDA model.

To study the PDA model, in addition to the four edge types (E1)–(E4) considered in Sect. 4, which partitions the set of pendant edges, we need two additional edge types concerning the internal edges. Specifically,

(E5): a type 5 edge is an internal edge adjacent to an independent cherry;
(E6): a type 6 edge is an internal edge that is not type 5.

For $1\le i \le 6$, let $E_i(T)$ be the set of edges of type *i*. Then the edge sets $E_1(T),\dots ,E_6(T)$ form a partition of the edge set of *T*. That is, each edge in *T* belongs to one and only one $E_i(T)$. Furthermore, let $\beta (T)=\big (|E_1(T)|, \dots ,|E_6(T)|\big )$ be the type vector associated with *T*, where $|E_i(T)|$ counts the number of type *i* edges in *T*.

**Fig. 3:**
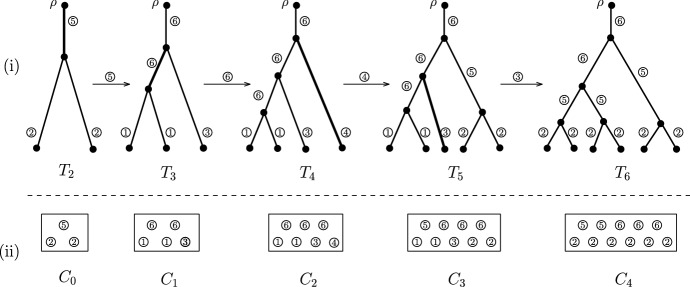
A sample path of the PDA model and the associated urn model. (i) A sample path of the PDA model evolving from $T_2$ with two leaves to $T_6$ with six leaves. The labels of the leaves are omitted for simplicity. The edge types are indicated by circled numbers. For $2\le i \le 5$, the edge selected in $T_i$ to generate $T_{i+1}$ is highlighted in bold and the associated edge type is indicated in the circled number above the arrows. (ii) The associated urn model with six colours, derived from the edge types in the trees. Note that in the vector form we have $C_0=(0,2,0,0,1,0), \ldots , C_3=(2, 2, 1, 0,1,3)$, and $C_4=(0,6,0,0,2,3)$

As illustrated in Fig. 3, the composition of edge types in *T*[*e*], which is obtained from *T* by attaching an extra leaf to edge *e*, is determined by the composition of edge types in *T* and the type of *e*. First, if *e* is a pendant edge, the change of the composition of the pendant edge types in *T*[*e*] is the same as described in Sect. 4, and the change of the composition of the interior edge types in *T*[*e*] is described as follows:

(i) If *e* is type 1, then $|E_i(T[e])|-|E_i(T)|$ is 0 if $i=5$, and 1 if $i=6$;
(ii) if *e* is type 2, then $|E_i(T[e])|-|E_i(T)|$ is $-1$ if $i=5$, and 2 if $i=6$;
(iii) if *e* is type 3, then $|E_i(T[e])|-|E_i(T)|$ is 2 if $i=5$, and $-1$ if $i=6$;
(iv) if *e* is type 4, then $|E_i(T[e])|-|E_i(T)|$ is 1 if $i=5$, and 0 if $i=6$.

Finally, when *e* is type 5, the change it caused is the same of that of a type 2 edge, and when *e* is type 6, the change it caused is the same of that of type 1 edge. Therefore, we can associate a PDA process starting with a tree $T_0$ with a corresponding urn process $(C_0,R)$ as follows. The urn model contains six colours in which colour *i* ($1\le i \le 6$) is designated for type *i* edges. In the initial urn $C_0=(C_{0,1},\dots ,C_{0,6})$, the number $C_{0,i}$ is precisely the number of type *i* edges in $T_0$. Furthermore, the replacement matrix *R* is the following $6 \times 6$ matrix:

16 $$\begin{aligned} R = \left[ \begin{array}{rrrrrr} 0~&{}0 ~&{}0 ~&{}1 ~&{}0 ~&{}1\\ 2~&{}-2~&{}1 ~&{}0 ~&{}-1 ~&{}2\\ -2~&{}4~&{}-1 ~&{}0 ~&{}2 ~&{}-1\\ 0~&{}2~&{}0 ~&{}-1 ~&{}1 ~&{}0\\ 2~&{}-2~&{}1 ~&{}0 ~&{}-1 ~&{}2\\ 0~&{}0~&{}0 ~&{}1 ~&{}0 ~&{}1 \end{array}\right] . \end{aligned}$$

Note that the replacement matrix for the YHK model in (8) is a submatrix of the replacement matrix in (16); and the last (respectively, second last) row in (16) is the same as its first (respectively, second) row. These two observations are direct consequences of the dynamic described above. The theorem below describes the asymptotic behaviour of $\beta (T_n)$, which enables us to deduce the asymptotic properties of the joint distribution of the number of pitchforks and the number of cherries for the PDA model in Corollary 2. Moreover, it also implies that the asymptotic behaviour of these two statistics, when appropriately scaled, is independent of the initial tree used in the PDA process.

### Theorem 4

Suppose that $T_m$ is an arbitrary phylogenetic tree with *m* leaves with $m\ge 2$, and that $T_n$ is a tree with *n* leaves generated by the PDA process starting with $T_m$.

Then we have

17 $$\begin{aligned} \frac{\beta (T_n)}{n} \xrightarrow {~a.s.~}{\mathbf {v}}_1 ~\quad ~\text{ and }~\quad ~ \frac{ \beta (T_n) - n{\mathbf {v}}_1 }{\sqrt{n}} \xrightarrow {~d~} {\mathcal {N}}\left( {\mathbf {0}},\varSigma \right) , \end{aligned}$$

as $n \rightarrow \infty$, where ${\mathbf {v}}_1= \frac{1}{16}(2,2,1,3,1,7)$ and

18 $$\begin{aligned} \varSigma = \frac{1}{64} \left[ \begin{array}{rrrrrr} 12 ~&{} -12 ~&{}6 ~&{}-6 ~&{} -6 ~&{}6\\ -12 ~&{} 28 ~&{}-6 ~&{}-10 &{} 14 ~&{} -14 \\ 6 ~&{} -6 ~&{}3 &{}-3 ~&{}-3 ~&{}3 \\ -6 ~&{} -10 ~&{}-3 ~&{} 19 ~&{}-5 ~&{}5 \\ -6 ~&{} 14 ~&{}-3 ~&{}-5 ~&{}7~&{} -7\\ 6 ~&{}-14 ~&{} 3 ~&{} 5 ~&{} -7 ~&{}7 \end{array}\right] . \end{aligned}$$

### Proof

Consider the PDA process $\{T_{n}\}_{n\ge m}$ starting with $T_m$. Let $C_{k}=\beta (T_{k+m})$ for $k\ge 0$. Then $C_k = (C_{k,1}, \dots , C_{k,6})$, where $C_{k,i}=|E_i(T_{m+k})|$ for $1\le i \le 6$, is the urn model of 6 colours derived from the edge partition of the PDA process. Therefore, it is a tenable model with $C_0=\beta (T_m)$ and replacement matrix *R* as given in (16).

Note that *R* is diagonalisable as

$$\begin{aligned}U^{-1}R U=\varLambda \end{aligned}$$

holds with $\varLambda =\text {diag}(2,0,0,0,-2,-4)$ and

19 $$\begin{aligned} U = \left[ \begin{array}{rrrrrr} 1 ~&{} 2.5 ~&{}2 ~&{}1 ~&{}1 ~&{}1\\ 1~&{}-2~&{}1 ~&{}0 ~&{}1 ~&{}5\\ 1 ~&{}-8 ~&{} -1 ~&{}1 ~&{} -3 ~&{} -9\\ 1 ~&{} -1 ~&{} 1 ~&{} 1 ~&{} -3 ~&{} -5 \\ 1 ~&{} 3 ~&{}-1 ~&{} 1 ~&{} 1 ~&{} 5 \\ 1 ~&{} 1 ~&{} -1 ~&{} -1 ~&{} 1 ~&{} 1 \end{array}\right] ~\quad ~\text{ and }~\quad ~ U^{-1} = \frac{1}{176}\left[ \begin{array}{rrrrrr} 22 ~&{} 22 ~&{} 11 ~&{} 33 ~&{} 11 ~&{} 77 \\ 4 ~&{} -20 ~&{} -14 ~&{} 14 ~&{} 6 ~&{} 10\\ 30 ~&{} 26 ~&{} -17 ~&{} 17 ~&{} -43 ~&{} -13 \\ 40 ~&{} -24 ~&{} 36 ~&{}-36 ~&{} 60 ~&{} -76\\ 66 ~&{} -22 ~&{} 33 ~&{} -77 ~&{}-11 ~&{} 11\\ -22 ~&{} 22 ~&{}-11 ~&{}11 ~&{} 11 ~&{}-11 \end{array}\right] . \end{aligned}$$

Therefore, *R* satisfies condition (A4). Next, (A2) holds because *R* has eigenvalues (counted with multiplicity)

$$\begin{aligned}s=\lambda _1=2,~\quad ~ \lambda _2=0, ~\quad ~\lambda _3=0, ~\quad ~\lambda _4=0,~\quad ~\lambda _5=-2,~\quad ~\lambda _6=-4\end{aligned}$$

where $s=\lambda _1=2$ is the principal eigenvalue. Furthermore, put ${\mathbf {u}}_i=U{\mathbf {e}}^\top _i$ and ${\mathbf {v}}_i={\mathbf {e}}_iU^{-1}$ for $1\le i \le 6$. Then (A3) follows by noting that ${\mathbf {u}}_1=(1,1,1,1,1,1)^\top$ is the principal right eigenvector, and ${\mathbf {v}}_1= \frac{1}{16}(2,2,1,3,1,7)$ is the principal left eigenvector.

The remainder of the proof is similar to the final part of the proof of Theorem 3, and hence we only outline the main steps. Since (A1)–(A4) are satisfied by the replacement matrix *R*, by Theorem 1 it follows that

$$\begin{aligned} \frac{C_k}{k} \xrightarrow {~a.s.~}{\mathbf {v}}_1 ~~\text{ with }~~ {k\rightarrow \infty ,} ~~\text{ and } \text{ hence }~~~ \frac{\beta (T_n)}{n}=\frac{n-m}{n} \frac{C_{n-m}}{n-m} \xrightarrow {~a.s.~}{\mathbf {v}}_1 ~~\text{ with }~~ {n\rightarrow \infty }. \end{aligned}$$

By Theorem 2 we have

20 $$\begin{aligned} \frac{ C_{n-m} - (n-m){\mathbf {v}}_1 }{\sqrt{n-m}}= \dfrac{ C_k - k v_1}{\sqrt{k}} \xrightarrow {~d~} {\mathcal {N}}({\mathbf {0}}, \varSigma ), \end{aligned}$$

where

21 $$\begin{aligned} \varSigma = \sum _{i,j=2}^6 \frac{ \lambda _i \lambda _j {{\mathbf {u}}}_i^\top \text{ diag }({\mathbf {v}}_1) {{\mathbf {u}}}_j }{1-\lambda _i -\lambda _j} {\mathbf {v}}_i^\top {\mathbf {v}}_j. \end{aligned}$$

Therefore, we have

$$\begin{aligned} \frac{ \beta (T_n) - n{\mathbf {v}}_1 }{\sqrt{n}} =&\frac{ C_{n-m} - (n-m){\mathbf {v}}_1 }{\sqrt{n}}+\frac{m{\mathbf {v}}_1}{\sqrt{n}} =\frac{\sqrt{n-m}}{\sqrt{n}}\frac{ C_{n-m} - (n-m){\mathbf {v}}_1 }{\sqrt{n-m}} \\&+\frac{m{\mathbf {v}}_1}{\sqrt{n}} \xrightarrow {~d~} {\mathcal {N}}\left( {\mathbf {0}},\varSigma \right) . \end{aligned}$$

$$\square $$

Similar to Corollary 1, by Theorem 4 it is straightforward to obtain the following result on the joint distribution of cherries and pitchforks.

### Corollary 2

Under the PDA model, for the joint distribution $(A_n,B_n)$ of pitchforks and cherries we have

22 $$\begin{aligned} \frac{1}{n}(A_n, B_n) \xrightarrow {~a.s.~}\Big (\frac{1}{8}, \frac{1}{4} \Big ) \end{aligned}$$

and

23 $$\begin{aligned} \frac{ (A_n, B_n) - n (1/8, 1/4)}{\sqrt{n}} \xrightarrow {~d~} {\mathcal {N}}\left( {\mathbf {0}},~ \frac{1}{64} \left[ \begin{array}{rr} 3 ~&{}~ 0 \\ 0 ~&{}~ 4 \end{array}\right] \right) \end{aligned}$$

as $$ n \rightarrow \infty $$.

### Proof

Consider the PDA process $\{T_{n}\}_{n\ge 2}$ starting with a tree $T_2$ with two leaves. Denote the *i*-th entry in $\beta (T_n)$ by $\beta _{n,i}$ for $1\le i \le 6$. Then the corollary follows from Theorem 3 by noting that we have $A_n=\frac{\beta _{n,1}}{2}$ and $B_n=\frac{\beta _{n,1}+\beta _{n,2}}{2}$. $\square$

The above result is consistent with the previously known results on the mean and (co-)variance of the joint distribution of cherries and pitchforks (see, e.g., Wu and Choi (2016); Choi et al. (2020)), namely, under the PDA model and for $n\ge 7$ we have

$$\begin{aligned} {\mathbb {E}}(A_n)&=\frac{n(n-1)(n-2)}{2(2n-3)(2n-5)}, ~~~~~~\quad {\mathbb {E}}(B_n)=\frac{n(n-1)}{2(2n-5)}, ~~&{\mathbb {V}}(B_n)=\frac{n(n-1)(n-2)(n-3)}{2(2n-3)^2(2n-7)}, \\ {\mathbb {V}}(A_n)&=\frac{3(4n^3-40n^2+123n-110)}{2(2n-5)(2n-7)(2n-9)} \, {\mathbb {V}}(B_n), ~~~~~\text{ and }~&Cov(A_n,B_n)=\frac{-{\mathbb {V}}(B_n)}{(2n-7)}. \end{aligned}$$

## Unrooted trees

Although rooted phylogenetic trees are often preferred by biologists as time is explicitly shown, it is also important to consider unrooted phylogenetic trees. Indeed, many methods for building trees from real data can usually do so only up to the placement of the root, and thus produce unrooted trees first and then figure out the root position (see, e.g. Steel (2016, Section 1.3)). In this section, we extend our results in Sects. 4 and 5 to unrooted phylogenetic trees.

**Fig. 4:**
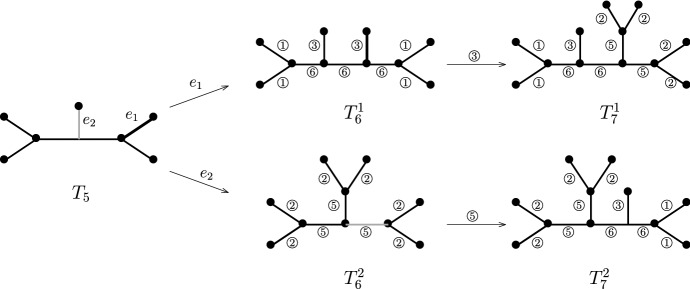
Example of sample paths for the PDA process on unrooted trees and the associated urn model. Two sample paths of the PDA process evolving from $T_5$: one ends with $T^1_7$ using the edges in bold and the other with $T^2_7$ using the edges in grey. Leave labels are omitted for simplicity. Note that in the vector form we have $\beta (T^1_6)=(4,0,2,0,0,3)$ and $\beta (T^2_6)=(0,6,0,0,3,0)$

Formally, deleting the root $\rho$ of a rooted phylogenetic tree and suppressing its adjacent interior vertex *r* results in an unrooted tree (see Fig. 4). The set of unrooted phylogenetic trees on $\{1,2,\dots ,n\}$ is denoted by ${\mathcal {T}}'_n$. The YHK process on unrooted phylogenetic tree is similar to that on rooted ones stated in Sect. 2.2; the only difference is that at step (ii) we shall start with an unrooted phylogenetic tree $T_m$ in ${\mathcal {T}}'_m$ for $m\ge 3$. Similar modification suffices for the PDA processes on unrooted phylogenetic trees; see Choi et al. (2020) for more details. Note that the concepts of cherries and pitchforks can be naturally extended to unrooted trees in ${\mathcal {T}}'_n$ for $n\ge 6$. Moreover, let $A'_n$ and $B'_n$ be the random variables counting the number of pitchforks and cherries in a random tree in ${\mathcal {T}}'_n$.

To associate urn models with the two processes on unrooted trees, note that for a tree *T* in ${\mathcal {T}}'_n$ with $n\ge 6$, we can decompose the edges in *T* into the six types similar to those for rooted trees, and hence define $\alpha (T)$ and $\beta (T)$ correspondingly. Furthermore, the replacement matrix is the same as the unrooted one, that is, the replacement matrix for the YHK model is given in (8) and the one for the PDA process is given in (16). See two examples in Fig. 4. We emphasize that the condition $n\ge 6$ is essential here: for instance, there is no appropriate assignment for the edge $e_2$ in the tree $T_5$ in Fig. 4 in our scheme, neither type 3 nor type 4 satisfying the requirement of a valid urn model. This observation is indeed in line with the treatment of unrooted trees in Choi et al. (2020). However, there is only one unrooted shape for $n=4$ and one for $n=5$. Furthermore, there are only two tree shapes for ${\mathcal {T}}'_6$ (as depicted in $T_6^1$ and $T_6^2$ in Fig. 4). In particular, putting $\alpha _6^1=(4,0,2,0)$ and $\alpha _6^2=(0,6,0,0)$, then for each *T* in ${\mathcal {T}}'_6$, we have either $\alpha (T)=\alpha _6^1$ or $\alpha (T)=\alpha _6^2$.

Now we extend Theorem 3 and Corollary 1 to the following result concerning the limiting behaviour of the YHK process. Similar to the rooted version, the asymptotic behaviour of the frequencies of cherries and pitchforks, when appropriately scaled, is independent of the initial trees used in the unrooted YHK process.

### Theorem 5

Suppose that $T_m$ is an arbitrary unrooted phylogenetic tree with *m* leaves with $m\ge 6$, and that $T_n$ is an unrooted tree with *n* leaves generated by the YHK process starting with $T_m$. Then, as $n \rightarrow \infty$,

24 $$\begin{aligned} \frac{\alpha (T_n)}{n} \xrightarrow {~a.s.~}{\mathbf {v}}_1 ~\quad ~\text{ and }~\quad ~ \frac{ \alpha (T_n) - n{\mathbf {v}}_1 }{\sqrt{n}} \xrightarrow {~d~} {\mathcal {N}}\left( {\mathbf {0}},\varSigma \right) , \end{aligned}$$

where ${\mathbf {v}}_1=\big (\frac{1}{3},\frac{1}{3},\frac{1}{6}, \frac{1}{6} \big )$ and $\varSigma$ is given in Eq. (10). In particular, as $n \rightarrow \infty$,

25 $$\begin{aligned} \frac{1}{n}(A'_n, B'_n) \xrightarrow {~a.s.~}\Big (\frac{1}{6}, \frac{1}{3} \Big ) ~\quad ~\text{ and }~\quad ~ \frac{ (A'_n, B'_n) - n (1/6, 1/3)}{\sqrt{n}} \xrightarrow {~d~} {\mathcal {N}}\left( {\mathbf {0}},\frac{1}{1260 } \left[ \begin{array}{rr} 69 ~&{} -28\\ -28 ~&{} 56 \end{array}\right] \right) . \end{aligned}$$

### Proof

The proof of (24) follows an argument similar to that for Theorem 4.

To establish (25), consider the YHK process $\{T_{n}\}_{n\ge 2}$ starting with a tree $T_2$ with two leaves. For $n\ge 6$, let $\alpha _n=\alpha (T_n)$ and $\alpha _{n,i}$ denote the *i*-th entry in $\alpha (T_n)$ for $1\le i \le 4$. Consider the vector $\alpha _6^1=(4,0,2,0)$ and $\alpha _6^2=(0,6,0,0)$. For $j=1,2$, let $E_j$ be the event that $\alpha _6=\alpha _6^j$. It follows that $E_1$ and $E_2$ form a partition of the sample space. Moreover, we have ${\mathbb {P}}(E_1)=4/5$ and ${\mathbb {P}}(E_2)=1-{\mathbb {P}}(E_1)=1/5$. Consider the random indicator variable ${\mathbb {I}}_{E_1}$, that is, ${\mathbb {P}}({\mathbb {I}}_{E_1}=1)=4/5$ and ${\mathbb {P}}({\mathbb {I}}_{E_1}=0)=1/5$. Random indicator variable ${\mathbb {I}}_{E_2}$ is similarly defined. Then we have

$$\begin{aligned} \alpha _n=\alpha ^1_n{\mathbb {I}}_{E_1}+\alpha ^2_n {\mathbb {I}}_{E_2}. \end{aligned}$$

Furthermore, by (24) we have $\frac{\alpha ^j_n}{n} \xrightarrow {~a.s.~}{\mathbf {v}}_1$ a.s. on $E_j$, for $j=1,2$, and hence

$$\begin{aligned} \frac{\alpha _n}{n} \xrightarrow {~a.s.~}{\mathbf {v}}_1 ({\mathbb {I}}_{E_1}+{\mathbb {I}}_{E_2})={\mathbf {v}}_1. \end{aligned}$$

Together with $A_n'=\frac{\alpha _{n,1}}{2}$ and $B_n'=\frac{\alpha _{n,1}+\alpha _{n,2}}{2}$, the almost surely convergence in (25) follows. Finally, the convergence in distribution in (25) also follows from a similar argument. $\square$

Finally, combining Theorem 4, Corollary 2, and an argument similar to the proof of Theorem 5 leads to the following result concerning the limiting behaviour of the unrooted PDA process, whose proof is hence omitted.

### Theorem 6

Suppose that $T_m$ is an arbitrary unrooted phylogenetic tree with *m* leaves with $m\ge 6$, and that $T_n$ is an unrooted tree with *n* leaves generated by the PDA process starting with $T_m$.

Then, as $$n \rightarrow \infty $$,

26 $$\begin{aligned} \frac{\beta (T_n)}{n} \xrightarrow {~a.s.~}{\mathbf {v}}_1 ~\quad ~\text{ and }~\quad ~ \frac{ \beta (T_n) - n{\mathbf {v}}_1 }{\sqrt{n}} \xrightarrow {~d~} {\mathcal {N}}\left( {\mathbf {0}},\varSigma \right) , \end{aligned}$$

where ${\mathbf {v}}_1= \frac{1}{16}(2,2,1,3,1,7)$ and $\varSigma$ is given in Eq. (18). In particular, as $n \rightarrow \infty$,

27 $$\begin{aligned} \frac{1}{n}(A'_n, B'_n) \xrightarrow {~a.s.~}\Big (\frac{1}{8}, \frac{1}{4} \Big ) ~~\quad ~\text{ and }~\quad ~ \frac{ (A'_n, B'_n) \!-\! n (1/8, 1/4)}{\sqrt{n}} \xrightarrow {~d~} {\mathcal {N}}\left( {\mathbf {0}},~ \frac{1}{64} \left[ \begin{array}{rr} 3 ~&{}~ 0 \\ 0 ~&{}~ 4 \end{array}\right] \right) . \end{aligned}$$

## Proofs of Theorems 1 and 2

In this section, we shall present the proofs of Theorems 1 and 2. To this end, it is more natural to consider $Y_n: = C_nU$, a linear transform of $C_n$. Next we introduce

28 $$\begin{aligned} \xi _n = Y_n - {\mathbb {E}}[Y_n \vert {{{\mathcal {F}}}}_{n-1}]. \end{aligned}$$

For $1 \le j \le d$, consider the following numbers

29 $$\begin{aligned} b_{n, n}(j) =1~~\text{ and } \quad b_{n, k}(j) = \prod _{\ell =k}^{n-1} (1+ \lambda _j/t_{\ell }) ~~~ \text{ for } 0 \le k <n. \end{aligned}$$

Moreover, we introduce the following diagonal matrix for $0\le k \le n$:

30 $$\begin{aligned} {\mathbf {B}}_{n,k}= \text {diag}\left( b_{n, k}(1), \ldots , b_{n,k}(d)\right) . \end{aligned}$$

Then we have the following key observation:

31 $$\begin{aligned} Y_n = Y_0 {\mathbf {B}}_{n,0} + \sum _{k=1}^n \xi _k\, {\mathbf {B}}_{n,k}. \end{aligned}$$

To see that (31) holds, let $Q_k = {\mathbf {I}} + t_{k-1}^{-1} R$ for $1 \le k \le n$, where ${\mathbf {I}}$ is the identity matrix. Then we have

$$\begin{aligned} {\mathbb {E}}[C_n \vert {{{\mathcal {F}}}}_{n-1}] = C_{n-1} + t_{n-1}^{-1} C_{n-1} R = C_{n-1} \left[ {\mathbf {I}} +t_{n-1}^{-1} R \right] = C_{n-1} Q_n.\end{aligned}$$

As $C_k - {\mathbb {E}}[C_k \vert {{{\mathcal {F}}}}_{k-1}]=\xi _k U^{-1}$ for $1\le k \le n$, we have

32 $$\begin{aligned} C_n&=(C_n - {\mathbb {E}}[C_n \vert {{{\mathcal {F}}}}_{n-1}]) + C_{n-1}Q_n =\xi _n U^{-1} + C_{n-1}Q_n \nonumber \\&=C_0 (Q_1\cdots Q_n)+ \xi _nU^{-1} +\sum _{k=1}^{n-1} \xi _k U^{-1} (Q_{k+1}\cdots Q_n). \end{aligned}$$

Since

33 $$\begin{aligned} U^{-1} \Big (\prod _{\ell =k+1}^n Q_{\ell } \Big ) U = \prod _{\ell =k}^{n-1} \big (U^{-1} \left( I+ t^{-1}_{\ell }R \right) U \big ) = \prod _{\ell =k}^{n-1} \left( I+ t_{\ell }^{-1} \varLambda \right) = {\mathbf {B}}_{n,k} \end{aligned}$$

holds for $0\le k \le n$ and $Y_n=C_nU$, it is straightforward to see that (31) follows from transforming (32) by a right multiplication of *U*.

Next, we shall present several properties concerning $\xi _k$. To this end, consider the sequence of random vectors $\tau _k=\chi _k-{\mathbb {E}}[\chi _k|{{{\mathcal {F}}}}_{k-1}]$ for $k\ge 1$. Then $\{\tau _k\}_{k\ge 1}$ is a martingale difference sequence (MDS) in that ${\mathbb {E}}[\tau _k|{{{\mathcal {F}}}}_{k-1}]={\mathbf {0}}$ almost surely. Hence ${\mathbb {E}}[\tau _k]={\mathbb {E}}\big [{\mathbb {E}}[\tau _k|{{{\mathcal {F}}}}_{k-1}]\big ]={\mathbf {0}}$. Furthermore, since the entries in $\chi _k$ are either 0 or 1 and ${\mathbb {E}}[\chi _k|{{{\mathcal {F}}}}_{k-1}]={\widetilde{C}}_{k-1}$, the random vector $\tau _k$ is also bounded. As a bounded martingale difference sequence, $\tau _k$ is uncorrelated. To see it, assuming that $\ell <k$, then we have

$$\begin{aligned} {\mathbb {E}}[\tau ^\top _\ell \tau _k ] ={\mathbb {E}}\big [{\mathbb {E}}[\tau ^\top _\ell \tau _k |{{{\mathcal {F}}}}_{k-1}] \big ] ={\mathbb {E}}\big [\tau ^\top _\ell {\mathbb {E}}[ \tau _k |{{{\mathcal {F}}}}_{k-1}] \big ] ={\mathbb {E}}[\tau ^\top _\ell {\mathbf {0}}] ={\mathbf {0}}^\top {\mathbf {0}}, \end{aligned}$$

where the first equality follows from the total law of expectation and the second from $\tau _{\ell }$ is ${\mathcal {F}}_{k-1}$-measurable. A similar argument shows ${\mathbb {E}}[\tau _\ell \tau ^\top _k ]=0$. Consequently, we have the following expression showing that distinct $\tau _k$ and $\tau _\ell$ are uncorrelated:

34 $$\begin{aligned} {\mathbb {E}}[\tau ^\top _k \tau _\ell ]={\mathbf {0}}^\top {\mathbf {0}}~~\text{ and }~~{\mathbb {E}}[\tau _k \tau ^\top _\ell ]=0~~\quad \text{ if } k\not = \ell . \end{aligned}$$

Moreover, putting

$$\begin{aligned}\varGamma _{k}: = \text {diag}\big ({\widetilde{C}}_{k}\big ) - {\widetilde{C}}_{k}^\top {\widetilde{C}}_{k},\end{aligned}$$

then we have

$$\begin{aligned}{\mathbb {E}}[\varGamma _{k}] = \text {diag}\big ({\mathbb {E}}[{\widetilde{C}}_{k}]\big ) - {\mathbb {E}}\big [ {\widetilde{C}}_{k}^\top {\widetilde{C}}_{k}\big ].\end{aligned}$$

Consequently, we have

35 $$\begin{aligned} {\mathbb {E}}[\tau ^\top _k \tau _k | {{{\mathcal {F}}}}_{k-1}]= & {} {\mathbb {E}}[ \big (\chi _k-{\mathbb {E}}[\chi _k|{{{\mathcal {F}}}}_{k-1}]\big )^\top \big (\chi _k-{\mathbb {E}}[\chi _k|{{{\mathcal {F}}}}_{k-1}]\big ) | {{{\mathcal {F}}}}_{k-1}] \nonumber \\= & {} {\mathbb {E}}[ \big (\chi ^\top _k-{\widetilde{C}}_{k-1}^\top \big ) \big (\chi _k-{\widetilde{C}}_{k-1}\big ) | {{{\mathcal {F}}}}_{k-1}] \nonumber \\= & {} {\mathbb {E}}[\chi ^\top _k\chi _k|{{{\mathcal {F}}}}_{k-1}] -{\widetilde{C}}_{k-1}^\top {\mathbb {E}}[\chi _k|{{{\mathcal {F}}}}_{k-1}] -{\mathbb {E}}[\chi _k^\top |{{{\mathcal {F}}}}_{k-1}]{\widetilde{C}}_{k-1} +{\widetilde{C}}_{k-1}^\top {\widetilde{C}}_{k-1} \nonumber \\= & {} {\mathbb {E}}[\chi ^\top _k\chi _k|{{{\mathcal {F}}}}_{k-1}]- {\widetilde{C}}_{k-1}^\top {\widetilde{C}}_{k-1} =\varGamma _{k-1}, \end{aligned}$$

where the last equality follows from (2). This implies

36 $$\begin{aligned} {\mathbb {E}}[\tau ^\top _k \tau _k] ={\mathbb {E}}\big [{\mathbb {E}}[\tau ^\top _k \tau _k | {{{\mathcal {F}}}}_{k-1}] \big ] ={\mathbb {E}}[\varGamma _{k-1}]. \end{aligned}$$

Note that $\xi _k$ is a ‘linear transform’ of $\tau _k$ in that combining (1) and (28) leads to

37 $$\begin{aligned} \xi _k= & {} \big (C_k - {\mathbb {E}}[C_k \vert {{{\mathcal {F}}}}_{k-1}] \big )U =\big (C_{k-1}+\chi _kR - {\mathbb {E}}[C_{k-1}+\chi _kR \vert {{{\mathcal {F}}}}_{k-1}] \big )U \nonumber \\= & {} \big (\chi _{k} - {\mathbb {E}}[\chi _k \vert {{{\mathcal {F}}}}_{k-1}] \big )RU =\tau _kRU =\tau _kU\varLambda . \end{aligned}$$

Note this implies that $\xi _k$ is a martingale difference sequence in that ${\mathbb {E}}[\xi _k|{{{\mathcal {F}}}}_{k-1}]={\mathbf {0}}={\mathbb {E}}[\xi _k]$. Furthermore, by (35) and (37) we have

38 $$\begin{aligned} {\mathbb {E}}\big [\xi _k^\top \xi _k|{{{\mathcal {F}}}}_{k-1}\big ]=\varLambda U^\top \varGamma _{k-1} U\varLambda ~\quad ~ \text{ for } k\ge 1. \end{aligned}$$

Together with (34) and (36), for all $k,\ell \ge 1$ we have

39 $$\begin{aligned} {\mathbb {E}}[\xi _k^\top \xi _k]=\varLambda U^\top {\mathbb {E}}[\varGamma _{k-1}] U\varLambda , \quad \text{ and } \quad {\mathbb {E}}[\xi _k^\top \xi _\ell ]={\mathbf {0}}^\top {\mathbf {0}}~ \text{ if } k\not =\ell . \end{aligned}$$

Since ${\mathbf {u}}_1=U {\mathbf {e}}^\top _1 ={\mathbf {e}}^\top$ is a right eigenvector of *R* corresponding to *s*, by (37) we have

40 $$\begin{aligned} \xi _k{\mathbf {e}}^\top _1 = \tau _kRU {\mathbf {e}}^\top _1 =\tau _kR{\mathbf {u}}_1 =s\tau _k{\mathbf {u}}_1 =s\tau _k{\mathbf {e}}^\top =0~\text{ for } k\ge 1, \end{aligned}$$

where the last equality follows from $\chi _k {\mathbf {e}}^\top =1$ and ${\mathbb {E}}[\chi _k|{{{\mathcal {F}}}}_{k-1}]{\mathbf {e}}^\top ={\widetilde{C}}_{k-1}{\mathbf {e}}^\top =1$.

Note that for $n>1$ and $\rho <1$, we have

41 $$\begin{aligned} \frac{1}{n}\sum _{k=1}^{n-1} \left( \frac{n}{k} \right) ^\rho \le \frac{1}{1-\rho }, ~~\text{ and }~~ \lim _{n\rightarrow \infty } \frac{1}{n} \sum _{k=1}^n \left( \frac{n}{k} \right) ^{\rho } = \int _0^1 x^{-\rho } dx = \frac{1}{1-\rho }. \end{aligned}$$

Furthermore, we present the following two results on the entries of ${\mathbf {B}}_{n,k}$, whose proofs are elementary calculus and included in the appendix.

### Lemma 1

Under assumptions (A2) and (A3), there exists a constant *K* such that

42 $$\begin{aligned} |b_{n,0}(j)|\le Kn^{\lambda _j/s} ~\quad ~\text{ and }~\quad ~ |b_{n,k}(j)| \le K(n/k)^{\lambda _j/s} \end{aligned}$$

hold for $1\le j \le d$ and $1\le k \le n$. Furthermore, we have

43 $$\begin{aligned} \lim _{n\rightarrow \infty } \frac{1}{n} \sum _{k=1}^n b_{n,k}(i) b_{n,k}(j) = \frac{s}{s- \lambda _i -\lambda _j} ~\quad \quad ~ \text{ for } 2\le i\le j \le d. \end{aligned}$$

### Corollary 3

Assume that $\{Z_n\}$ is a sequence of random variables such that

$$\begin{aligned} Z_n \xrightarrow {~1~}Z \end{aligned}$$

for a random variable *Z*. Then under assumptions (A2)-(A3), for $2\le i \le j \le d$ we have

44 $$\begin{aligned} \frac{1}{n}\sum _{k=1}^n b_{n,k}(i)b_{n,k}(j)Z_k \xrightarrow {~p~}\frac{s}{s- \lambda _i -\lambda _j} Z ~\quad ~ \text{ as } n\rightarrow \infty . \end{aligned}$$

### Proof of Theorem 1

#### Proof

Recall that $Y_n = C_nU$ for $n\ge 1$. Hence, it is sufficient to show that

45 $$\begin{aligned} n^{-1} Y_n \xrightarrow {~a.s.~}s\, {\mathbf {e}}_1 \end{aligned}$$

because $s\, {\mathbf {e}}_1U^{-1}= s\, {\mathbf {v}}_1$ and $n^{-1}C_n=n^{-1}Y_nU^{-1}$. Furthermore, as the sequence of random vectors $n^{-1}C_n$ is bounded, its $L^r$ convergence follows from the almost sure convergence.

To establish (45), we restate the following decomposition from  (31) as below:

46 $$\begin{aligned} Y_n = Y_0 {\mathbf {B}}_{n,0} + \sum _{k=1}^n \xi _k\, {\mathbf {B}}_{n,k}, \end{aligned}$$

where $\{\xi _k\}$ is the martingale difference sequence in (28) and ${\mathbf {B}}_{n,k}$ is the diagonal matrix in (30).

Next we claim that

47 $$\begin{aligned} n^{-1}{\mathbb {E}}[Y_n]\longrightarrow s\, {\mathbf {e}}_1~~\text{ as } n\rightarrow \infty . \end{aligned}$$

Indeed, since ${\mathbb {E}}[\xi _k]={\mathbf {0}}$ implies ${\mathbb {E}}[\xi _k\, {\mathbf {B}}_{n,k}]={\mathbb {E}}[\xi _k]{\mathbf {B}}_{n,k}={\mathbf {0}}$, by (46) we have ${\mathbb {E}}[Y_n]=Y_0 {\mathbf {B}}_{n,0}.$ Therefore the *j*-th entry in ${\mathbb {E}}[Y_n]$, denoted by $y_{n,j}$, is given by

$$\begin{aligned} y_{n,j}= {\mathbb {E}}[Y_n]{\mathbf {e}}_j^\top =Y_0 {\mathbf {B}}_{n,0} {\mathbf {e}}_j^\top =b_{n,0}(j) Y_0 {\mathbf {e}}_j^\top ~\text{ for } 1\le j \le d. \end{aligned}$$

When $j=1$, we have

$$\begin{aligned} y_{n,1}=b_{n,0}(1) Y_0 {\mathbf {e}}_1^\top =(t_n/t_0)Y_0 {\mathbf {e}}_1^\top =(t_n/t_0)C_0 U {\mathbf {e}}_1^\top =(t_n/t_0)C_0 {\mathbf {u}}_1 =(t_n/t_0)t_0=t_n, \end{aligned}$$

where we used the fact that ${\mathbf {u}}_1={\mathbf {e}}^\top$ and hence $t_0=C_0 {\mathbf {u}}_1$. Therefore we have $y_{n,1}/n=t_n/n \rightarrow s$ as $n\rightarrow \infty$. On the other hand, for $2\le j \le d$ there exist two constants $K_1$ and *K* such that

$$\begin{aligned} |y_{n,j}|=|b_{n,0}(j) Y_0 {\mathbf {e}}_j^\top | \le K_1|b_{n,0}(j)| \le K_1K n ^{\lambda _j/s}, \end{aligned}$$

where the last inequality follows from Lemma 1. Since $\lambda _j<s$, it follows that $y_{n,j}/n \rightarrow 0$ as $n\rightarrow \infty$. This completes the proof of (47).

For simplicity, let $Z_{n}:=Y_{n} - {\mathbb {E}}(Y_{n})$. Then we have $Y_n=Z_n+{\mathbb {E}}(Y_n)$, by (47) it follows that to establish (45), it remains to show that

48 $$\begin{aligned} Z_n/n \xrightarrow {~a.s.~}{\mathbf {0}}, \end{aligned}$$

Denote the *j*-th entry in $Z_n$ by $Z_{n,j}$, then from (46) we have

49 $$\begin{aligned} Z_{n,j} =\sum _{k=1}^n (\xi _k {\mathbf {B}}_{n,k}){\mathbf {e}}^\top _j = \sum _{k=1}^n b_{n,k}(j) \xi _k {\mathbf {e}}^\top _j. \end{aligned}$$

Since (48) is equivalent to

50 $$\begin{aligned} \frac{Z_{n,j}}{n} \xrightarrow {~a.s.~}0 ~~~~~~~~ \text{ for } 1\le j \le d, \end{aligned}$$

the remainder of the proof is devoted to establishing (50).

It is straightforward to see that (50) holds for $j=1$ because by (40) and (49) we have

$$\begin{aligned} Z_{n,1}=\sum _{k=1}^n b_{n,k}(j) \xi _k {\mathbf {e}}^\top _1 =0. \end{aligned}$$

Thus in the remainder of the proof, we may assume that $2\le j \le d$ holds. Note that

$$\begin{aligned} {\mathbb {E}}\big [ Z_{n,j}^2\big ]= & {} {\mathbb {E}}\bigg [\bigg (\sum _{k=1}^n b_{n,k}(j) \xi _k {\mathbf {e}}^\top _j \bigg )^2 \bigg ] = {\mathbb {E}}\big [ \sum _{k,\ell =1}^n b_{n,k}(j) b_{n,\ell }(j) {\mathbf {e}}_j \xi ^\top _k \xi _{l} {\mathbf {e}}^\top _j \big ] \\= & {} {\mathbb {E}}\big [ \sum _{k=1}^n b^2_{n,k}(j) {\mathbf {e}}_j \xi _k^\top \xi _{k} {\mathbf {e}}^\top _j \big ] = \sum _{k=1}^n b^2_{n,k}(j) {\mathbb {E}}\big [{\mathbf {e}}_j \xi _k^\top \xi _{k} {\mathbf {e}}^\top _j \big ]. \end{aligned}$$

Here the third equality follows from (39). As ${\mathbb {E}}[{\mathbf {e}}_j \xi ^\top _k \xi _{k} {\mathbf {e}}^\top _j]$, the (*j*, *j*)-entry of matrix ${\mathbb {E}}[\xi ^\top _k \xi _{k}]$, is bounded above by a constant $K_1$ in view of (39), there exist constants $K_2$ and *K* so that

$$\begin{aligned} {\mathbb {E}}\big [ Z_{n,j}^2\big ]&\le K_1 \sum _{k=1}^n |b_{n,k}(j)|^2 \le K_2 \ \sum _{k=1}^n \left( \frac{n}{k} \right) ^{2 \lambda _{j}/s} =K_2+ K_2n \ \sum _{k=1}^{n-1} \frac{1}{n}\left( \frac{k}{n} \right) ^{-2 \lambda _{j}/s} \\&\le K_2+ \frac{K_2n}{1-2 \lambda _{j}/s} \le Kn \end{aligned}$$

holds for all $n\ge 1$. Here the second inequality follows from Lemma 1 and the third one from (41) in view of $\lambda _j < s/2$ for $2 \le j \le d$.

Since ${\mathbb {E}}(Z_{n,j})=0$, for $\epsilon >0$ using the Chebychev inequality we get

51 $$\begin{aligned} {\mathbb {P}}\left( \left| Z_{n,j} \right| >n \epsilon \right) \le \frac{K}{n \epsilon ^2} ~\quad \text{ for } \text{ all } n\ge 1. \end{aligned}$$

Consider the subsequence $Z'_{n,j}$ of $Z_{n,j}$ with $Z'_{n,j}=Z_{n^2, j}$ for $n \ge 1$. Then for $\epsilon >0$ we have

$$\begin{aligned} \sum _{n=1}^\infty {\mathbb {P}}\left( \frac{|Z'_{n,j}|}{n^2}> \epsilon \right) = \sum _{n=1}^\infty {\mathbb {P}}\left( \left| Z_{n^2,j}\right| > n^2\epsilon \right) \le \sum _{n=1}^\infty \frac{K }{n^2 \epsilon ^2} <\infty , \end{aligned}$$

where the first inequality follows from (51). Thus, by the Borel-Cantelli Lemma, it follows that

52 $$\begin{aligned} {n^{-2}}{Z'_{n,j}} \xrightarrow {~a.s.~}0. \end{aligned}$$

Next, consider

$$\begin{aligned} \varDelta _{n,j}&:= \max _{n^2 \le k< (n+1)^2 } | Z_{k,j} -Z'_{n,j} | = \max _{n^2 \le k < (n+1)^2 } | Z_{k,j} -Z_{n^2,j} | = \max _{1 \le k \le 2n } |Z_{n^2+k,j} -Z_{n^2,j} |. \end{aligned}$$

Since for each $\ell > 0$, elements of $\chi _\ell$ and *RU* are all bounded above, there exists a constant *K* independent of $\ell$ and *j* so that

$$\begin{aligned} |Z_{\ell +1,j}-Z_{\ell ,j}|&= |\big ((C_{\ell +1}-{\mathbb {E}}[C_{\ell +1}])-(C_{\ell }-{\mathbb {E}}[C_{\ell }])\big )U {\mathbf {e}}^\top _j| \\&=|\big (C_{\ell +1}-C_{\ell }\big )-\big ({\mathbb {E}}[C_{\ell +1}-C_{\ell }]\big )U {\mathbf {e}}^\top _j| = |\big (\chi _{\ell +1}-{\mathbb {E}}[\chi _{\ell +1}] \big ) RU{\mathbf {e}}^\top _j| \le K. \end{aligned}$$

Consequently, we have

$$\begin{aligned} \varDelta _{n,j}&=\max _{0 \le k \le 2n } |Z_{n^2+k,j} -Z_{n^2,j} | \le \max _{1 \le k \le 2n } \sum _{\ell =1}^k |Z_{n^2+\ell ,j} -Z_{n^2+\ell -1,j} | \le \max _{1 \le k \le 2n } \sum _{\ell =1}^k K =2nK, \end{aligned}$$

and hence

53 $$\begin{aligned} n^{-2}{\varDelta _{n,j}} \xrightarrow {~a.s.~}0. \end{aligned}$$

Now, for each $k>0$, considering the natural number *n* with $n^2 \le k < (n+1)^2$, then we have

54 $$\begin{aligned} \frac{\left| Z_{k,j}\right| }{k} \le \frac{\left| Z_{k,j} -Z_{n^2,j}\right| }{k} +\frac{\left| Z_{n^2,j}\right| }{k} \le \frac{\varDelta _{n,j}}{n^2}+\frac{\left| Z_{n^2,j}\right| }{n^2}= \frac{\varDelta _{n,j}}{n^2}+\frac{\left| Z'_{n,j}\right| }{n^2}. \end{aligned}$$

Note that when $k\rightarrow \infty$, the natural number *n* satisfying $n^2 \le k < (n+1)^2$ also approaches to $\infty$. Thus combining (52), (53), and (54) leads to

55 $$\begin{aligned} k^{-1}{Z_{k,j}} \xrightarrow {~a.s.~}0 \qquad \text {when }k\rightarrow \infty , \end{aligned}$$

which completes the proof of (50), and hence also the theorem. $\square$

### Proof of Theorem 2

#### Proof

For each $n\ge 1$, consider the following two sequences of random vectors:

$$\begin{aligned} X_{n,k}: = n^{-1/2} \xi _k {\mathbf {B}}_{n,k} \quad ~\text{ and } \quad S_{n,k}: = \sum _{\ell =1}^k X_{n,\ell } ~~\quad ~~\text{ for } 1\le k \le n, \end{aligned}$$

where $\{\xi _k\}_{k\ge 1}$ is the martingale difference sequence in (28) and ${\mathbf {B}}_{n,k}$ is the diagonal matrix in (30). Then for each $n\ge 1$, the sequence $\{X_{n,k}\}_{1\le k \le n}$ is a martingale difference sequence, and $\{S_{n,k}\}_{1\le k \le n}$ is a mean zero martingale.

Recalling that $Y_n = C_nU$, then by (31) we have

56 $$\begin{aligned} S_{n,n}= n^{-1/2}\sum _{k=1}^n \xi _k {\mathbf {B}}_{n,k} = n^{-1/2}\big (Y_n - {\mathbb {E}}[Y_n ] \big ). \end{aligned}$$

A key step in our proof is to show that

57 $$\begin{aligned} S_{n,n} \xrightarrow {~d~} {\mathcal {N}}({{\mathbf {0}}}, {\widetilde{\varSigma }}), \end{aligned}$$

where ${\mathcal {N}}({{\mathbf {0}}}, {\widetilde{\varSigma }})$ denotes a normal distribution with mean vector ${\mathbf {0}}$ and variance-covariance matrix

58 $$\begin{aligned} {\widetilde{\varSigma }}: = \ \sum _{i, j=2}^d \frac{s\lambda _i \lambda _j {\mathbf {u}}_i^\top \text {diag}({\mathbf {v}}_1) {\mathbf {u}}_j }{s-\lambda _i -\lambda _j} {\mathbf {e}}_i^\top {\mathbf {e}}_j. \end{aligned}$$

We shall show that Theorem 2 follows from  (57). To this end, we claim that

59 $$\begin{aligned} Z_n:= n^{-1/2} \left( {\mathbb {E}}[Y_n]-ns {\mathbf {e}}_1 \right) \longrightarrow {\mathbf {0}} ~~ \text{ with } n\rightarrow \infty . \end{aligned}$$

Indeed, we have $Z_{n}{\mathbf {e}}^\top _1=n^{-1/2}(t_n- ns)=n^{-1/2}t_0 \rightarrow 0$. Furthermore, by Lemma 1 there exists a constant *K* such that

$$\begin{aligned} |Z_n{\mathbf {e}}^\top _j| = n^{-1/2}|Y_{0,j} b_{n,0}(j)|=n^{-1/2}Y_{0,j} |b_{n,0}(j)| \le n^{-1/2}Y_{0,j} K n ^{\lambda _j/s} ~~\quad ~~ \text{ for } 2\le j \le d. \end{aligned}$$

As $\lambda _j/s <1/2$, it follows that $|Z_{n}{\mathbf {e}}^\top _j| \rightarrow 0$ for all $1\le j \le d$, and hence (59) holds. Consequently, we have

60 $$\begin{aligned} n^{-1/2} \left( Y_n - ns {\mathbf {e}}_1\right) = n^{-1/2} \left( Y_n - {\mathbb {E}}[Y_n] \right) + Z_n =S_{n,n}+Z_n \xrightarrow {~d~} N({{\mathbf {0}}}, {\widetilde{\varSigma }}). \end{aligned}$$

Here the second equality follows from (56); convergence in distribution follows from the Slutsky theorem (see, e.g. Grimmett and Stirzaker (2001, P. 318)) in view of (57) and (59). Since $n^{-1/2} (C_n - ns {\mathbf {v}}_1) =n^{-1/2} \left( Y_n - ns {\mathbf {e}}_1\right) V$ with $V=U^{-1}$, by (60) and the fact that a linear transform of a normal vector is also normal (see, e.g. Grimmett and Stirzaker (2001, Section 4.9)), we have

61 $$\begin{aligned} n^{-1/2} (C_n - ns {\mathbf {v}}_1) \xrightarrow {~d~} N({{\mathbf {0}}}, \varSigma ), \end{aligned}$$

where

62 $$\begin{aligned} \varSigma = V^\top {{\widetilde{\varSigma }}} \, V=V^\top \Big (\sum _{i, j=2}^d \frac{s\lambda _i \lambda _j {\mathbf {u}}_i^\top \text {diag}({\mathbf {v}}_1) {\mathbf {u}}_j }{s-\lambda _i -\lambda _j} {\mathbf {e}}_i^\top {\mathbf {e}}_j \Big ) V =\sum _{i, j=2}^d \frac{s\lambda _i \lambda _j {\mathbf {u}}_i^\top \text {diag}({\mathbf {v}}_1) {\mathbf {u}}_j }{s-\lambda _i -\lambda _j} {\mathbf {v}}_i^\top {\mathbf {v}}_j, \end{aligned}$$

which shows indeed that the theorem follows from (57).

What remains is to prove  (57). Define

$$\begin{aligned} \varPhi (n):=\sum _{k=1}^n {\mathbb {E}}\big [X_{n,k}^\top X_{n,k} |{{{\mathcal {F}}}}_{k-1}\big ] =\frac{1}{n} \sum _{k=1}^n {\mathbf {B}}_{n,k} {\mathbb {E}}[ \xi _k ^\top \xi _k |{{{\mathcal {F}}}}_{k-1} ] {\mathbf {B}}_{n,k}. \end{aligned}$$

We next show that

63 $$\begin{aligned} \varPhi (n) \xrightarrow {~p~}{\widetilde{\varSigma }}. \end{aligned}$$

Let $\varGamma = \text {diag}({\mathbf {v}}_1) - {\mathbf {v}}_1^\top {\mathbf {v}}_1$. Note that for $2 \le i,j\le d$, we have ${\mathbf {v}}_1 {\mathbf {u}}_i = 0={\mathbf {v}}_1 {\mathbf {u}}_j$ in view of (4), and hence

$$\begin{aligned} \frac{s\lambda _i \lambda _j {\mathbf {u}}_i^\top \varGamma {\mathbf {u}}_j }{s- \lambda _i-\lambda _j} = \frac{s\lambda _i \lambda _j {\mathbf {u}}_i^\top (\text {diag}({\mathbf {v}}_1) - {\mathbf {v}}_1^\top {\mathbf {v}}_1) {\mathbf {u}}_j }{s- \lambda _i-\lambda _j} =\frac{s\lambda _i \lambda _j {\mathbf {u}}_i^\top \text {diag}({\mathbf {v}}_1) {\mathbf {u}}_j }{s- \lambda _i-\lambda _j}. \end{aligned}$$

Therefore (63) is equivalent to

64 $$\begin{aligned} {\mathbf {e}}_i \varPhi (n) {\mathbf {e}}_j^\top \xrightarrow {~p~}{\left\{ \begin{array}{ll} \dfrac{s\lambda _i \lambda _j \ {\mathbf {u}}_i^\top \varGamma {\mathbf {u}}_j}{s- \lambda _i-\lambda _j} \, &{}~ 2 \le i, j \le d, \\ 0 &{} ~ \text {if }i=1\text { or }j=1. \end{array}\right. } \end{aligned}$$

Since ${\mathbf {B}}_{n,k}$ is a diagonal matrix and ${\mathbf {e}}_1\xi _k^\top =0$ in view of (40), this implies

$$\begin{aligned} {\mathbf {e}}_1 \varPhi (n) =\frac{1}{n} \sum _{k=1}^n {\mathbf {e}}_1 {\mathbf {B}}_{n,k} {\mathbb {E}}[ \xi _k ^\top \xi _k \,|\, {{{\mathcal {F}}}}_{k-1}] {\mathbf {B}}_{n,k} =\frac{1}{n} \sum _{k=1}^n b_{n,k}(1) {\mathbb {E}}[ {\mathbf {e}}_1\xi _k ^\top \xi _k \,|\, {{{\mathcal {F}}}}_{k-1}] {\mathbf {B}}_{n,k} ={\mathbf {0}}. \end{aligned}$$

A similar argument shows $\varPhi (n) {\mathbf {e}}^\top _1={\mathbf {0}}$, and hence (64) holds for $i=1$ or $j=1$. It remains to consider the case $2\le i,j\le d$. Since

$$\begin{aligned} {\widetilde{C}}_k \xrightarrow {~1~}{\mathbf {v}}_1 ~~\quad \text{ and }~~\quad {\widetilde{C}}^\top _k{\widetilde{C}}_k \xrightarrow {~1~}{\mathbf {v}}^\top _1{\mathbf {v}}_1 \end{aligned}$$

hold in view of Theorem 1,

we have

65 $$\begin{aligned} \lambda _i \lambda _j {\mathbf {u}}_i^\top \varGamma _{k} {\mathbf {u}}_j \xrightarrow {~1~}\lambda _i \lambda _j {\mathbf {u}}_i^\top \varGamma {\mathbf {u}}_j ~~\text{ as } k \rightarrow \infty . \end{aligned}$$

As both ${\mathbf {B}}_{n, k}$ and $\varLambda$ are diagonal matrices, we have

66 $$\begin{aligned} \frac{1}{n}&\sum _{i=k}^n {\mathbf {e}}_i {\mathbf {B}}_{n,k} (\varLambda U^\top \varGamma _{k-1} U\varLambda ) {\mathbf {B}}_{n,k} \ {\mathbf {e}}^\top _j = \frac{1}{n} \sum _{k=1}^n b_{n,k}(i) b_{n,k}(j) {\mathbf {e}}_i\varLambda U^\top \varGamma _{k-1} U\varLambda {\mathbf {e}}^\top _j \nonumber \\&\quad = \frac{\lambda _i \lambda _j}{n} \sum _{k=1}^n b_{n,k}(i) b_{n,k}(j) {\mathbf {u}}_i^\top \varGamma _{k-1} {\mathbf {u}}_j \xrightarrow {~p~}\frac{s\lambda _i \lambda _j {\mathbf {u}}_i^\top \varGamma {\mathbf {u}}_j }{s- \lambda _i-\lambda _j}, \end{aligned}$$

where the convergence follows from Corollary 3 and (65).

Since $S_{n,n}$ is a mean ${\mathbf {0}}$ random vector and ${\mathbf {B}}_{n,k}$ is a diagonal matrix, we have

$$\begin{aligned} {\mathbb {V}}\left[ S_{n,n} \right]&={\mathbb {E}}[S^\top _{n,n}S_{n,n}] = \frac{1}{n} \sum _{k,\ell =1}^n {\mathbf {B}}^\top _{n,k} {\mathbb {E}}[ \xi _k ^\top \xi _\ell ] {\mathbf {B}}_{n,\ell } =\frac{1}{n} \sum _{k=1}^n {\mathbf {B}}_{n,k} {\mathbb {E}}[ \xi _k ^\top \xi _k ] {\mathbf {B}}_{n,k} \\&= \sum _{k=1}^n {\mathbb {E}}[X^\top _{n,k} X_{n,k}] ={\mathbb {E}}[\varPhi (n)] \end{aligned}$$

where the third equality follows from (39).

Furthermore, an argument similar to the proof of (63) shows that

$$\begin{aligned} \lim _{n\rightarrow \infty } {\mathbb {V}}(S_{n,n}) = {\widetilde{\varSigma }}. \end{aligned}$$

Therefore ${\widetilde{\varSigma }}$ is positive semi-definite because the matrix ${\mathbb {V}}(S_{n,n})$ is necessarily positive semi-definite for each $n\ge 1$.

Following the Cramér-Wold device for the multivariate central limit theorem (see, e.g. Durrett (2019, Theorem 3.10.6)), fix an arbitrary row vector ${\mathbf {w}}=(w_1,\dots ,w_{d})$ in ${\mathbb {R}}^{d}\setminus \{{\mathbf {0}}\}$ and put $s_{n,k}=S_{n,k} {\mathbf {w}}^\top$ and $x_{n,k}=X_{n,k}{\mathbf {w}}^\top$. Furthermore, since the matrix ${\widetilde{\varSigma }}$ is positive semi-definite, we can introduce $\sigma ^2:={\mathbf {w}}\, {\widetilde{\varSigma }}\, {\mathbf {w}}^\top \ge 0$. Then for establishing (57) it suffices to show that

67 $$\begin{aligned} s_{n,n} \xrightarrow {~d~} N(0, \sigma ^2). \end{aligned}$$

Since $\{x_{n,k}\}_{1\le k \le n}$ is a martingale difference sequence and $\{s_{n,k}\}_{1\le k \le n}$ is an array of mean zero martingale, the martingale central limit theorem (see, e.g. Hall and Heyde (2014, Corollary 3.2)) implies that (67) follows from

68 $$\begin{aligned} \gamma _n:= \sum _{k=1}^{n} {\mathbb {E}}\left[ \left| x_{n,k} \right| ^2 \vert {{{\mathcal {F}}}}_{k-1} \right] \xrightarrow {~p~}\sigma ^2 ~~\quad ~\text{ as } n\rightarrow \infty \end{aligned}$$

and the conditional Lindeberg-type condition holds, that is, for every $\epsilon >0$

69 $$\begin{aligned} \gamma ^*_n:=\sum _{k=1}^n {\mathbb {E}}\left[ \left| x_{n,k} \right| ^2 {\mathbb {I}}_{A_{n,k,\epsilon }} \vert {{{\mathcal {F}}}}_{k-1} \right] \xrightarrow {~p~}0 ~~\quad ~\text{ as } n\rightarrow \infty \end{aligned}$$

where ${\mathbb {I}}_{A_{n,k,\epsilon }}$ is the indicator variable on $A_{n,k,\epsilon }: = \{ |x_{n,k}| > \epsilon \}$.

Now (68) follows from

70 $$\begin{aligned} \gamma _n&= \sum _{k=1}^{n} {\mathbb {E}}\left[ {\mathbf {w}}X_{n,k}^\top X_{n,k}{\mathbf {w}}^\top |{{{\mathcal {F}}}}_{k-1} \right] = {\mathbf {w}}\sum _{k=1}^{n} {\mathbb {E}}\left[ X_{n,k}^\top X_{n,k} |{{{\mathcal {F}}}}_{k-1} \right] {\mathbf {w}}^\top \nonumber \\&= {\mathbf {w}}\,\varPhi (n)\,{\mathbf {w}}^\top \xrightarrow {~p~}{\mathbf {w}}\, {\widetilde{\varSigma }}\, {\mathbf {w}}^\top = \sigma ^2, \end{aligned}$$

where the convergence follows from (63).

To see that (69) holds, by (37) we have

$$\begin{aligned} X_{n,k}=\sum _{j=1}^{d} X_{n,k}{\mathbf {e}}^\top _j {\mathbf {e}}_j=\sum _{j=1}^{d} n^{-1/2} \lambda _j\,b_{n, k}(j) \tau _k {{\mathbf {u}}}_j {\mathbf {e}}_j, ~~\quad ~{1\le k \le n}. \end{aligned}$$

In particular, we have $X_{n,k}(1)=0$ because $\tau _k {{\mathbf {u}}}_1=0$ holds for $k\ge 1$ in view of (40). Consequently, we have

71 $$\begin{aligned} x_{n,k}=X_{n,k} {\mathbf {w}}^\top =\sum _{j=2}^{d} n^{-1/2} w_j \lambda _j b_{n, k}(j) \tau _k {{\mathbf {u}}}_j. \end{aligned}$$

Putting $\rho =\lambda _2/s$, then $\lambda _j/s\le \rho <1/2$ holds for $2\le j \le d$ in view of (A2) and (A4). Furthermore, there exists a constant $K_0>0$ independent of *n* and *k* such that

72 $$\begin{aligned} |x_{n,k}| \le \sum _{j=2}^{d} n^{-1/2} |w_j \lambda _j \tau _k {{\mathbf {u}}}_j| |b_{n, k}(j)| \le K_0 n^{-1/2}(n/k)^{\rho } \le K_0n^{-1/2}\max (1, n^{\rho }) \end{aligned}$$

holds for $1\le k \le n$. Here the second inequality follows from Lemma 1 and the fact that $| w_j \lambda _j \tau _k {\mathbf {u}}_j|$ is bounded above by a constant independent of *k*. The last inequality follows from the fact that $(n/k)^{\rho } \le \max \big ((n/1)^{\rho },(n/n)^{\rho } \big )$. Now let $A'_{n,\epsilon }: = \{ K_0n^{-1/2}\max (1, n^{\rho })> \epsilon \}$, which it is either $\emptyset$ if *n* is sufficient large or the whole probability space otherwise. Then by (72) we have $A_{n,k,\epsilon }\subseteq A'_{n,\epsilon }$ and hence for all $\epsilon >0$ and each *n*, we have ${\mathbb {I}}_{A_{n,k,\epsilon }}\le {\mathbb {I}}_{A'_{n,\epsilon }}$ for all $1\le k \le n$. Furthermore, since $\rho <1/2$ and $K_0>0$, we have

73 $$\begin{aligned} {\mathbb {E}}[{\mathbb {I}}_{A'_{n,\epsilon }}]={\mathbb {P}}(A'_{n,\epsilon }) \rightarrow 0~~\text{ as } n \rightarrow \infty . \end{aligned}$$

Consequently, we have

74 $$\begin{aligned} {\mathbb {E}}[\gamma ^*_n]&= {\mathbb {E}}\Big [ \sum _{k=1}^{n} {\mathbb {E}}\big [ \left| x_{n,k} \right| ^2 {\mathbb {I}}_{A_{n,k,\epsilon }} \vert {{{\mathcal {F}}}}_{k-1} \big ] \Big ] \le {\mathbb {E}}\Big [ \sum _{k=1}^{n} {\mathbb {E}}\big [ \left| x_{n,k} \right| ^2 {\mathbb {I}}_{A'_{n,\epsilon }} \vert {{{\mathcal {F}}}}_{k-1} \big ] \Big ] \end{aligned}$$

75 $$\begin{aligned}&= {\mathbb {E}}\Big [ \Big (\sum _{k=1}^{n} {\mathbb {E}}\big [ \left| x_{n,k} \right| ^2 \vert {{{\mathcal {F}}}}_{k-1} \big ] \Big ) {\mathbb {I}}_{A'_{n,\epsilon }} \Big ] ={\mathbb {E}}\left[ \gamma _n{\mathbb {I}}_{A'_{n,\epsilon }} \right] \end{aligned}$$

76 $$\begin{aligned}&= {\mathbb {E}}\big [ \gamma _n \big ] {\mathbb {E}}\big [ {\mathbb {I}}_{A'_{n,\epsilon }}\big ] \rightarrow 0,~~\text{ as } n\rightarrow \infty \end{aligned}$$

where we have used the fact that ${\mathbb {I}}_{A'_{n,\epsilon }}$ is ${{{\mathcal {F}}}}_{n}$-measurable and independent of ${{{\mathcal {F}}}}_{n}$ (and all its sub-sigma-algebras); the convergence follows from (70) and (73). Since $\gamma ^*_n$ is almost surely non-negative, this completes the proof of (69), the last step in the proof of the theorem. $\square$

## Discussion

Inspired by a martingale approach developed by Bai and Hu (2005), we present in this paper the strong law of large numbers and the central limit theorem for a family of the Pólya urn models in which negative off-diagonal entries are allowed in their replacement matrices. This leads to a unified approach to proving corresponding limit theorems for the joint vector of cherry and pitchfork counts under the YHK model and the PDA model. In other words, the results for both models are derived from Theorems 1 and 2, using different replacement matrices. Furthermore, our results on unrooted trees are also derived directly from Theorems 1 and 2, without the need for a detour of rooted trees. For each of these random tree models, we show that the joint vector of cherry and pitchfork frequencies converges almost surely to a deterministic vector and the appropriately scaled fluctuations converge in distribution to a bivariate normal distribution. Interestingly, such convergence results do not depend on the initial tree used in the generating process.

The results presented here also lead to several broad directions that may be interesting to explore in future work. The first direction concerns a more detailed analysis on convergence. For instance, the central limit theorems present here should be extendable to a functional central limit theorem (see, e.g. Gouet (1993)), a follow-up project that we will pursue. Furthermore, it remains to establish the rate of convergence for the limit theorems (see Laulin (2020) for some recent results on urns with two colours). For example, a law of the iterated logarithm would add considerable information to the strong law of large numbers by providing a more precise estimate of the size of the almost sure fluctuations of the random sequences in Theorems 3 and 4.

The second direction concerns whether the results obtained here can be extended to other tree statistics and tree models. For example, the two tree models considered here, the YHK and the PDA, can be regarded as special cases of some more general tree generating models, such as Ford’s alpha model (see, e.g. Chen et al. (2009)) and the Aldous beta-splitting model (see, e.g. Aldous (1996)). Therefore, it is of interest to extend our studies on subtree indices to these two models as well. Furthermore, instead of cherry and pitchfork statistics, we can consider more general subtree indices such as *k*-pronged nodes and *k*-caterpillars (Rosenberg 2006; Chang and Fuchs 2010).

Finally, it would be interesting to study tree shape statistics for several recently proposed graphical structures in evolutionary biology. For instances, one can consider aspects of tree shapes that are related to the distribution of branch lengths (Ferretti et al. 2017; Arbisser et al. 2018) or relatively ranked tree shapes (Kim et al. 2020). Furthermore, less is known about shape statistics in phylogenetic networks, in which non-tree-like signals such as lateral gene transfer and viral recombinations are accommodated (Bouvel et al. 2020). Further understanding of their statistical properties could help us design more complex evolutionary models that may in some cases provide a better framework for understanding real datasets.

## Appendix

In the appendix we present a proof of Lemma 1 and Corollary 3. To this end, we start with the following observation.

### Lemma 2

For $\lambda \in {\mathbb {R}}$, $\ell \in {\mathbb {R}}_{> 0}$, and two non-negative integers *m* and *n* with $n\ge m$, put

$$\begin{aligned} F_m^m(\ell , \lambda )=1, ~\quad ~ \text{ and } ~\quad ~ F_m^n(\ell , \lambda ):= \prod _{i=m}^{n-1} \left( 1+\frac{\lambda }{\ell + i} \right) ~\quad ~ \text{ for } n>m. \end{aligned}$$

Then we have

77 $$\begin{aligned} \lim _{m \rightarrow \infty } \sup _{n\ge m} \left( \frac{m}{n} \right) ^{\lambda } F_m^n(\ell , \lambda ) =1. \end{aligned}$$

Furthermore, there exists a positive constant $K=K(\lambda ,\ell )$ such that

78 $$\begin{aligned} \left| F_m^n(\ell , \lambda ) \right| \le K \left( n/m\right) ^\lambda ~~\text{ for } \text{ all } 1\le m\le n. \end{aligned}$$

### Proof

Since the lemma holds for $\lambda =0$ in view of $F_m^n(\ell ,0)=1$, we assume that $\lambda \not = 0$ in the remainder of the proof. For simplicity, put $L:=\max \big (1,-(\ell +\lambda )\big )$.

First we shall establish (77). To this end, we may assume $m>L$, and hence $m+\ell +\lambda >0$. Furthermore, recall the following result on the ratio of gamma functions: for a fixed number $y\in {\mathbb {R}}$, we have

79 $$\begin{aligned} \lim _{x\rightarrow \infty } \frac{\varGamma (x+y)}{x^y\varGamma (x)}=1, \end{aligned}$$

which follows from Stirling’s formula for the gamma function; see also Jameson (2013, P.398) for an alternative approach. Therefore, putting

$$\begin{aligned} G_{m,k}:=\frac{\varGamma (m+k+\ell +\lambda )}{(m+k)^\lambda \varGamma (m+k+\ell )} ~\quad ~\text{ for } \text{ integer } k\ge 0, \end{aligned}$$

then we have

80 $$\begin{aligned} \lim _{m\rightarrow \infty } \ln \big (G_{m,0}\big ) =0, ~\quad ~\text{ and } \text{ hence }~\quad ~ \lim _{m\rightarrow \infty } \sup _{k\ge 0} \ln \big (G_{m+k,0}\big )=0. \end{aligned}$$

Here the second limit holds because the limit of $\ln \big (G_{m,0}\big )$ being 0 implies that its limit superior is also 0. Together with $G_{m,k}=G_{m+k,0}$ for $k\ge 0$, this leads to

81 $$\begin{aligned} \lim _{m\rightarrow \infty } \sup _{k\ge 0} \ln \big (G_{m,k}\big ) = \lim _{m\rightarrow \infty } \sup _{k\ge 0} \ln \big (G_{m+k,0}\big ) =0. \end{aligned}$$

Since

82 $$\begin{aligned} \left( \frac{m}{m+k} \right) ^{\lambda } F_m^{m+k}(\ell , \lambda )&= \left( \frac{m}{m+k} \right) ^{\lambda } \frac{ \varGamma (m+k+\ell +\lambda )\varGamma (m+\ell )}{ \varGamma (m+k+\ell )\varGamma (m+\ell +\lambda )} =\frac{G_{m,k}}{G_{m,0}} \end{aligned}$$

holds for each integer $k\ge 0$, we have

$$\begin{aligned} \lim _{m \rightarrow \infty } \sup _{n\ge m} \ln \Big (\left( \frac{m}{n} \right) ^{\lambda } F_m^n(\ell , \lambda ) \Big )= & {} \lim _{m \rightarrow \infty } \sup _{k\ge 0} \ln \Big (\Big (\frac{m}{m+k} \Big )^{\lambda } F_m^{m+k}(\ell , \lambda ) \Big )\ \\= & {} \lim _{m \rightarrow \infty } \sup _{k\ge 0} \Big (\ln (G_{m,k})-\ln (G_{m,0})\Big ) \\= & {} \lim _{m \rightarrow \infty } \sup _{k\ge 0} \ln (G_{m,k})- \lim _{m \rightarrow \infty } \ln (G_{m,0})\\= & {} 0, \end{aligned}$$

where the last equality follows from  (80) and  (81). This completes the proof of (77).

Next, we shall establish (78). To this end we assume $m<n$, $m+\ell +\lambda \not =0$, and $n-1+\ell +\lambda \not =0$ as otherwise it clearly holds. Now consider the following three cases:

Case 1: $1\le m \le n-1 < L$, and hence $n-1+\ell +\lambda < 0$. Let $A=\{(\alpha ,\beta )\,| \alpha , \beta \in {\mathbb {N}}; 1\le \alpha \le \beta \le 1 -\ell -\lambda \}$ be the finite subset of ${\mathbb {N}}\times {\mathbb {N}}$ whose size depends on $\ell$ and $\lambda$, and consider the constant

$$\begin{aligned}K_1:=\max _{(\alpha ,\beta )\in A} \big \{|F_\alpha ^\beta (\ell ,\lambda )| \left( \alpha /\beta \right) ^\lambda \big \}. \end{aligned}$$

Since $(m,n)\in A$, it follows that $|F_m^n(\ell ,\lambda )|\le K_1(n/m)^\lambda$ holds.

Case 2: $m\ge L$ and hence $m+\ell +\lambda >0$. Note that in this case we have $F_m^n(\ell ,\lambda )>0$. Furthermore, an argument similar to the proof of  (77) shows that for each $m\ge L$ we have

$$\begin{aligned} \lim _{n \rightarrow \infty } \left( \frac{m}{n} \right) ^{\lambda } F_m^n(\ell , \lambda ) =\frac{1}{G_{m,0}}, \end{aligned}$$

and hence there exists a constant $K'_m$ depending on $m, \ell ,\lambda$ so that $F_m^n(\ell ,\lambda )\le K'_m (n/m)^\lambda$ holds. Furthermore, by (77) it follows that there exists a constant $M=M(\ell ,\lambda )$ and a constant $K_0=K_0(\ell ,\lambda )$ so that $F_m^n(\ell ,\lambda )\le K_0 (n/m)^\lambda$ holds for all $m> M$. Therefore, for the constant

$$\begin{aligned} K_2:=\max \{K_0,K'_1,\dots ,K'_{M} \}, \end{aligned}$$

which depends only on $\ell$ and $\lambda$, we have $F_m^n(\ell ,\lambda )\le K_2 (n/m)^\lambda$ for all $L\le m \le n$.

Case 3: $1\le m<L<n-1$ and hence $m+\ell +\lambda<0<n-1+\ell +\lambda$. Note this implies $L>1$ and we may further assume that *L* is not an integer as otherwise $F_m^n(\ell ,\lambda )=0$ follows. Let *p* be the (necessarily positive) largest integer less than *L*. Then $1\le m\le p<L$ and we have $|F_m^p(\ell ,\lambda )|\le K_1(p/m)^\lambda$ for a constant $K_1$ in view of Case 1 and the fact that $F_m^m(\ell , \lambda )=1$. Furthermore, as $p+1>L$, by Case 2 we have $|F_{p+1}^n(\ell ,\lambda )|\le K_2(n/p+1)^\lambda$. Therefore, considering the constant $K_3=\max \{K_1K_2,2^{-\lambda } K_1K_2\}$, which depends on only $\ell$ and $\lambda$, we have

$$\begin{aligned} |F_m^n(\ell ,\lambda )|= & {} |F_m^p(\ell ,\lambda ) F_{p+1}^n(\ell ,\lambda )| \le K_1K_2 \left( \frac{p}{m}\right) ^\lambda \left( \frac{n}{p+1}\right) ^\lambda \\= & {} K_1K_2 \left( \frac{p}{p+1}\right) ^\lambda \left( \frac{n}{m}\right) ^\lambda \le K_3 \left( \frac{n}{m}\right) ^\lambda . \end{aligned}$$

The last inequality follows since $(\frac{p}{p+1})^\lambda \le 1$ holds for $\lambda >0$, and $(\frac{p}{p+1})^\lambda \le 2^{-\lambda }$ for $\lambda <0$. $\square$

With Lemma 2, we now present a proof of Lemma 1.

### Proof of Lemma 1

Recall that by (A3) we have $t_{\ell }=t_0+\ell s$ for $\ell \ge 1$, and hence

$$\begin{aligned} b_{n, k}(j) = \prod _{\ell =k}^{n-1} \Big (1+ \frac{\lambda _j}{t_0+\ell s} \Big ) =\prod _{\ell =k}^{n-1} \Big (1+ \frac{\lambda _j/s}{(t_0/s)+\ell } \Big ) =F_k^n\Big (\frac{t_0}{s},\frac{\lambda _j}{s} \Big ) \end{aligned}$$

holds for $1\le j \le d$ and $1\le k < n$. Noting that $b_{n,n}(j)=1$, by Lemma 2 there exists a constant $K'_j$ such that $|b_{n,k}(j)|\le K'_j(n/k)^{\lambda _j/s}$ holds for $1\le k \le n$. Now let $a_j=1+(\lambda _j/t_0)$ and put $K_j=\max (K'_j,K'_j|a_j|)$. Then we have $|b_{n,0}(j)|\le K_jn^{\lambda _j/s}$ in view of $b_{n,0}(j)=a_jb_{n,1}(j)$. This establishes  (42) by choosing $K=\max (K_1,\dots ,K_d)$.

Next, we shall show (43). To this end, fix a pair of indices $2\le i \le j \le d$, and put $\rho _i=\lambda _i/s$ and $\rho _j=\lambda _j/s$. Then by (A2) we have $\rho :=\rho _i+\rho _j<1$ and $1-\rho =(s-\lambda _i-\lambda _j)/s$. Furthermore, consider

$$\begin{aligned} \varDelta _{n}:=\frac{1}{n}\sum _{k=1}^n \left( \frac{n}{k} \right) ^{\rho _j} \Big ( b_{n,k}(i)-\left( \frac{n}{k} \right) ^{\rho _i} \Big ) ~~ \text{ and } ~~ \varDelta ^*_{n}:=\frac{1}{n}\sum _{k=1}^n b_{n,k}(i) \Big ( b_{n,k}(j)-\left( \frac{n}{k} \right) ^{\rho _j} \Big ). \end{aligned}$$

Then we have

$$\begin{aligned} \varDelta _{n}+\varDelta ^*_{n}= \frac{1}{n}\sum _{k=1}^n \Big ( b_{n,k}(i)b_{n,k}(j)-\left( \frac{n}{k} \right) ^{\rho } \Big ). \end{aligned}$$

By (41) it suffices to show that both $\varDelta _{n}\rightarrow 0$ and $\varDelta ^*_{n} \rightarrow 0$ as $n\rightarrow \infty$.

By (42), we have $|b_{n,k}(i)| \le K(n/k)^{\rho _i}$ and $|b_{n,k}(j)| \le K(n/k)^{\rho _j}$. We shall first show that $\varDelta _{n} \rightarrow 0$ as $n\rightarrow \infty$. To this end, let

$$\begin{aligned} H_{n,k}:=\left( \frac{n}{k} \right) ^{\rho _j} \Big ( b_{n,k}(i)-\left( \frac{n}{k} \right) ^{\rho _i} \Big ) =\left( \frac{n}{k} \right) ^{\rho } \Big ( \Big (\frac{k}{n} \Big )^{\rho _i} b_{n,k}(i)-1 \Big ). \end{aligned}$$

Then we have $|H_{n,k}|\le (K+1) (n/k)^{\rho }$ for all $1\le k \le n$. Consider $\epsilon >0$. Then it follows that $\epsilon ':=\frac{\epsilon (1-\rho )}{2(2-\rho )}>0$. By (77) in Lemma 2, we have

$$\begin{aligned} \lim _{k \rightarrow \infty } \sup _{n\ge k} \left( \frac{k}{n} \right) ^{\rho _i} b_{n,k}(i) = \lim _{k \rightarrow \infty } \sup _{n\ge k} \left( \frac{k}{n} \right) ^{\rho _i} F_k^n\Big (\frac{t_0}{s}, \rho _i\Big ) =1. \end{aligned}$$

Therefore, there exists a positive integer *M* such that

$$\begin{aligned} \sup _{n\ge k} \left( \Big (\frac{k}{n} \Big )^{\rho _i} b_{n,k}(i)-1 \right) \le \epsilon ' \end{aligned}$$

holds for all $k\ge M$. Consequently, $|H_{n,k}|\le \epsilon ' (n/k)^{\rho }$ holds for all $n\ge k \ge M$. Moreover, let *N* be the smallest integer greater than *M* so that $N>[2(K+1)M/\epsilon ]^{1/(1-\rho )}$ and $N>M[2(K+1)/\epsilon ]^{1/(1-\rho )}$ both hold. Then for $n>N$ we have

$$\begin{aligned} \frac{1}{n}\sum _{k=1}^n |H_{n,k}|&\le \frac{\epsilon '}{n}\sum _{k=M+1}^n \left( \frac{n}{k} \right) ^{\rho } +\frac{1}{n}\sum _{k=1}^{M} |H_{n,k}| \le \frac{\epsilon '}{n}\sum _{k=1}^n \left( \frac{n}{k} \right) ^{\rho } +\frac{1}{n}\sum _{k=1}^{M} |H_{n,k}| \\&\le \frac{\epsilon '(2-\rho )}{1-\rho }+\frac{K+1}{n^{1-\rho }}\sum _{k=1}^{M} \left( \frac{1}{k} \right) ^{\rho } \le \frac{\epsilon }{2}+\frac{\epsilon }{2} = \epsilon , \end{aligned}$$

where in the third inequality we use the fact that (41) implies

$$\begin{aligned} \frac{1}{n}\sum _{k=1}^n \left( \frac{n}{k} \right) ^{\rho }\le \frac{1}{n}+\frac{1}{1-\rho }\le 1+\frac{1}{1-\rho }=\frac{2-\rho }{1-\rho }. \end{aligned}$$

Therefore it follows that $\varDelta _{n} \rightarrow 0$ as $n \rightarrow \infty$. Since $|b_{n,k}(i)| \le K(n/k)^{\rho _i}$, a similar argument can be adopted to show that $\varDelta ^*_{n} \rightarrow 0$ as $n \rightarrow \infty$, completing the proof of Lemma 1. $\square$

Finally, we complete the appendix by the following proof of Corollary 3.

### Proof of Corollary 3

Fix a pair of indexes $2\le i \le j \le d$. For simplicity, we put $a_{n,k}=b_{n,k}(i)b_{n,k}(j)$. Furthermore, let $\rho =(\lambda _i+\lambda _j)/s$, then $\rho <1$ and $1-\rho =(s-\lambda _i-\lambda _j)/s>0$. Then by Lemma 1 we have

83 $$\begin{aligned} \lim _{n\rightarrow \infty } \frac{1}{n}\sum _{k=1}^n a_{n,k}=\frac{1}{1-\rho }, ~\quad ~ \text{ and } ~\quad ~ |a_{n,k}| \le K\Big (\frac{n}{k}\Big )^\rho ~\quad ~ ~\text{ for } \text{ all } n\ge 1 \text{ and } 1\le k\le n. \end{aligned}$$

Furthermore, let $N_0$ be the smallest integer greater than 1 such that both $N_0> -(\lambda _i+t_0)/s$ and $N_0>-(\lambda _j+t_0)/s$ hold. Then we have $a_{n,k}>0$ for all $n\ge k \ge N_0$.

We shall next show that

84 $$\begin{aligned} \frac{1}{n} \sum _{k=1}^n a_{n,k} {\mathbb {E}}[|Z_k-Z|] \rightarrow 0. \end{aligned}$$

For simplicity, put $\beta _k:={\mathbb {E}}[|Z_k-Z|]$ for $k\ge 1$. Then $\{\beta _k\}_{k\ge 1}$ is a sequence of non-negative numbers which converges to 0. Thus there exists a constant $K_1>0$ such that $\beta _k<K_1$ holds for all $k\ge 1$. Next, fix an arbitrary number $\epsilon >0$. By (83), let $N_1=N_1(\epsilon )$ be the smallest integer greater than $N_0$ so that

85 $$\begin{aligned} \frac{1}{n}\sum _{k=1}^n a_{n,k}<\frac{1}{1-\rho }+\epsilon ~ ~\text{ for } \text{ holds } \text{ for } \text{ all } n>N_1. \end{aligned}$$

Since $1-\rho > 0$, the number $\epsilon ':=\frac{\epsilon (1-\rho )}{2(1+\epsilon (1-\rho ))}$ is greater than 0. Let $N_2$ be the smallest positive integer greater than $N_1$ so that $\beta _k<\epsilon '$ holds for all $k>N_2$. Now let *N* be the smallest positive integer greater than $N_2$ so that $N\ge (2(K_1+\epsilon ')KN_2/\epsilon )^{1/(1-\rho )}$ and $N\ge N_2(2(K_1+\epsilon ')K/\epsilon )^{1/(1-\rho )}$ both hold. Then for $n>N$ we have

$$\begin{aligned} \Big | \frac{1}{n} \sum _{k=1}^n a_{n,k}\beta _k \Big |&\le \Big | \frac{1}{n} \sum _{k=1}^{N_2} a_{n,k}\beta _k\Big |+\frac{1}{n} \sum _{k=1+N_2}^n a_{n,k}\beta _k \le \frac{K_1}{n} \sum _{k=1}^{N_2} |a_{n,k}|+\frac{\epsilon '}{n} \sum _{k=1+N_2}^n a_{n,k}\\&= \frac{K_1}{n} \sum _{k=1}^{N_2} |a_{n,k}|-\frac{\epsilon '}{n}\sum _{k=1}^{N_2} a_{n,k} + \frac{\epsilon '}{n} \sum _{k=1}^n a_{n,k} \\&\le \frac{K_1+\epsilon '}{n} \sum _{k=1}^{N_2} |a_{n,k}|+\frac{\epsilon '}{n} \sum _{k=1}^n a_{n,k}\\&\le \frac{(K_1+\epsilon ')KN_2\max (n^{\rho },(n/N_2)^{\rho })}{n}+\frac{\epsilon '}{n} \sum _{k=1}^n a_{n,k} \\&\le \frac{\epsilon }{2}+\epsilon '\Big (\frac{1}{1-\rho }+\epsilon \Big ) = \epsilon , \end{aligned}$$

from which (84) follows. Here the first inequality follows from the triangle inequality and that $a_{n,k}\beta _k>0$ holds for $n\ge k >N_2\ge N_0$, the second inequality holds since $0\le \beta _k<K_1$ for $k\ge 1$ and $\beta _k<\epsilon '$ for $k>N_2$. Next, the third inequality holds since we have $\epsilon '(a_{n,k}+|a_{n,k}|) \ge 0$ for $1\le k \le n$. Furthermore, the fourth inequality holds because by (83) we have $|a_{n,k}|\le K\max (n^{\rho },(n/N_2)^{\rho })$ for $1\le k \le N_2$, and the last inequality follows from (85) and that $2(K_1+\epsilon ')KN_2n^\rho \le \epsilon n$ and $2(K_1+\epsilon ')KN^{1-\rho }_2n^\rho \le \epsilon n$ hold in view of $n>N$.

Finally, by (83) and (84) it follows that

$$\begin{aligned} \frac{1}{n}\sum _{k=1}^n a_{n,k}Z \xrightarrow {~p~}\frac{1}{1-\rho }Z ~\quad ~\text{ and }~\quad ~ \frac{1}{n} \sum _{k=1}^n a_{n,k} (Z_k-Z) \xrightarrow {~p~}0. \end{aligned}$$

Therefore, we can conclude that

$$\begin{aligned} \frac{1}{n} \sum _{k=1}^n a_{n,k}Z_k=\frac{1}{n}\sum _{k=1}^n a_{n,k} Z+ \frac{1}{n}\sum _{k=1}^n a_{n,k} (Z_k-Z) \xrightarrow {~p~}\frac{1}{1-\rho }Z, \end{aligned}$$

as required. $$\square $$
